# Design principles for rapid folding of knotted DNA nanostructures

**DOI:** 10.1038/ncomms10803

**Published:** 2016-02-18

**Authors:** Vid Kočar, John S. Schreck, Slavko Čeru, Helena Gradišar, Nino Bašić, Tomaž Pisanski, Jonathan P. K. Doye, Roman Jerala

**Affiliations:** 1 Department of Biotechnology, National Institute of Chemistry, Hajdrihova 19, Ljubljana 1000, Slovenia; 2 Graduate school of Biomedicine, University of Ljubljana, Ljubljana 1000, Slovenia; 3 Department of Chemistry, Physical and Theoretical Chemistry Laboratory, University of Oxford, South Parks Road, Oxford OX1 3QZ, UK; 4 EN-FIST, Centre of Excellence, Trg Osvobodilne fronte 13, Ljubljana 1000, Slovenia; 5 Faculty of Mathematics and Physics, University of Ljubljana, Jadranska 19, Ljubljana 1000, Slovenia; 6 FAMNIT, University of Primorska, Glagoljaška 8, Koper 6000, Slovenia

## Abstract

Knots are some of the most remarkable topological features in nature. Self-assembly of knotted polymers without breaking or forming covalent bonds is challenging, as the chain needs to be threaded through previously formed loops in an exactly defined order. Here we describe principles to guide the folding of highly knotted single-chain DNA nanostructures as demonstrated on a nano-sized square pyramid. Folding of knots is encoded by the arrangement of modules of different stability based on derived topological and kinetic rules. Among DNA designs composed of the same modules and encoding the same topology, only the one with the folding pathway designed according to the ‘free-end' rule folds efficiently into the target structure. Besides high folding yield on slow annealing, this design also folds rapidly on temperature quenching and dilution from chemical denaturant. This strategy could be used to design folding of other knotted programmable polymers such as RNA or proteins.

## Abstract (web summary)

Driven by complementary base pairing, artificial single-chain DNA is capable of forming complex 3D architectures if an appropriate folding pathway can be realised. Here, the authors describe the design principles for rapidly folding structures, exemplified through fabrication of a nanosized square pyramid.

## Graphical abstract

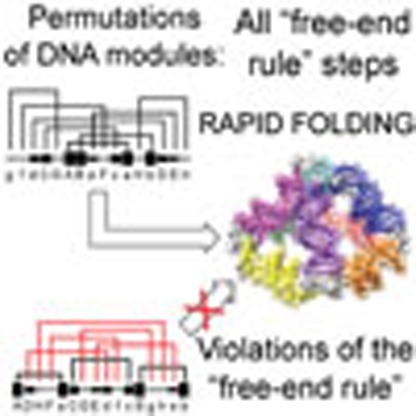

Natural biopolymers are able to fold into well-defined complex three-dimensional structures, as determined by the information encoded in the linear polymer sequence^1^. Programmable characteristics of DNA, RNA and proteins have long been recognized as a means for the design and self-assembly of functional bionanostructures^2^. Besides the need for the target structure to possess favourable thermodynamic properties, kinetic features of the energy landscape, which affect not only the rate of folding but also the ability to attain complex folds, should be considered^3^. One such example is the formation of knots, which are intricate topological features with important natural and technological implications. Knotted polymers have significant technological potential similar to that in the macroscopic materials, where knots underlie the mechanical properties of textiles. Knots are found in nucleic acids and proteins^4^. Knotted proteins are quite rare and occur most frequently in the form of a trefoil (3_1_) knot or a slipknot^5^, although recently a protein with a crossing number of six (forming a Stevedore's 6_1_ knot) has been identified^6^,^7^. Knots enhance thermal^8^,^9^ and mechanical^9^ stability of proteins but are challenging to fold^10^.

Owing to its programmable nature, predictability of its stability and straightforward synthesis^11^,^12^, DNA is an almost ideal prototyping material for designing nanostructures^13^. To date, a plethora of structures, from small topological constructs^14^,^15^ to megadalton two-dimensional^16^,^17^,^18^ and three-dimensional^19^,^20^,^21^,^22^,^23^ assemblies, has been reported. An important constraint for the folding of knotted single-chain DNA polyhedra^24^,^25^ is threading of knots^26^, which requires the correct order of folding steps^27^. Although thermodynamic optimization works well for simpler structures, robust design principles to control folding of increasingly complex single-chain DNA polyhedra, which could be extended to alternative biopolymers (for example, RNA and proteins), are needed.

Here we define rules to guide the folding of highly knotted single-chain DNA nanostructures based on defining the order of formation of duplex modules according to their stability. A ‘free-end' rule states that at each folding step the favourable folding design must involve at least one pairing segment with a free end, allowing its threading through the preformed structure. We prove that such a design is theoretically always possible. Coarse-grained simulations demonstrated the validity of this rule, and that threading of long tails through small loops is kinetically hindered. Folding of DNA square pyramids composed of the same modules and encoding the same topology revealed that only the design with the folding pathway designed according to the ‘free-end' rule folds efficiently into the target structure. Besides high folding yields on slow annealing, this design also folds rapidly on temperature quenching and dilution from chemical denaturant.

## Results

### Considerations for the design of folding pathways

**Figure 1:**
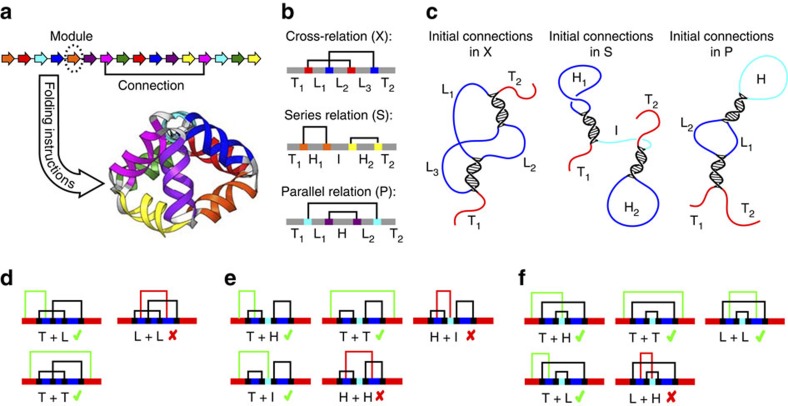
The free end rule as a topological determinant for the folding of polyhedral single-chain DNA nanostructures. (**a**) The linear arrangement of orthogonal, pairwise-interacting modules defines the polyhedral structure of the target fold. Colour-coded pairs of complementary modules (arrows) bind to form connections that make up the double-helical edges of the pyramid. (**b**) Possible elementary relations between two connections are depicted. (**c**) Based on distinct topological relations, pairing between the initial connections can lead to the formation of knots (X), two independent hairpins (S) and hairpins with internal loops (P). Unstructured terminal segments (T) are depicted in red; hairpin loops (H) and internal loops (L) are depicted in blue; an internal unstructured segment (I) is depicted in cyan. (**d**–**f**) Favoured versus unfavoured connections following the initial connections are depicted for situations, when the initial connections are in a cross (**d**), series (**e**) or parallel relation (**f**).

Our approach to devise design principles for folding knotted single-chain biopolymers was based on the identification of folding steps that are topologically and kinetically favourable, and on the use of the designable stability of the pairwise interacting modules, which provides the means to steer the folding. First, we identified the favourable and the unfavourable steps in the folding of modular nanostructures (Fig. 1). Three elementary topological relations between any two connections that link a pair of complementary modules have been defined^28^: a cross (X), series (S) or parallel (P) (Fig. 1b). Depending on the initial pair of connections, each remaining module can be classified into a terminus (T), an internal loop (L), a hairpin loop (H) or an internal segment (I) (Fig. 1c). Whether or not the remaining free complementary modules are able to pair correctly in the consecutive folding steps depends on the previously formed connections, in particular when the length of the modules exceeds one helical turn (∼10.5 bp), which leads to the formation of knots. Pairing of segments between loops is topologically unfavourable, as both strands are fixed at the ends and are therefore unable to freely wrap around each other, to form a full-length double helix (for example, L+L, H+H and L+H in Fig. 1d–f, respectively). Instead, only less stable kissing loops can be formed^29^,^30^, unless some previously formed connections are disrupted. Moreover, certain connections (for example, I+H in Fig. 1e) are unfavourable due to kinetic rather than topological restrictions, as they require threading of the chain with previously formed loops through another loop. We therefore define that a folding pathway is favourable when it is composed of connection-forming steps that involve at least one terminal module (type T), which we call the ‘free-end' rule. Importantly, it can be proven that for every single-chain polyhedron with edges formed of dimeric modules, at least two folding pathways can be designed where all steps comply with the ‘free-end' rule (Supplementary Note 1).

### Single-chain DNA pyramid topology and the design of modules

To guide the folding pathways of DNA nanostructures, we can exploit the designable stability and orthogonality (that is, full-length connections only form between complementary segments) of individual modules. The feasibility of this approach is demonstrated on the single-chain DNA square pyramid (4 Py; Fig. 1a), which is the simplest convex polyhedron composed of antiparallel duplex edges^31^. Out of the five theoretically possible double Eulerian circuits (that is, cyclic paths in connected graphs that visit each edge exactly twice in an antiparallel orientation)^31^,^32^, we selected a circuit where the chain does not cross itself at the apex (Supplementary Fig. 1a). Eight pairs of complementary 14-mer sequences were designed as the building modules for the construction of edges of the 4 Py, which results in a structure with a crossing number of 35 (ref. 33). Stabilities of each pair were determined from melting curves of hairpin oligonucleotides (Supplementary Fig. 2 and Supplementary Table 1).

### Theoretical assessment of designed folding pathways

To set the ‘free-end' rule on a more quantitative foundation and analyse the kinetics of each connection-forming step, simulations were performed using the coarse-grained oxDNA program based model^34^,^35^. To calculate the rates and sample transition path ensembles of successive connection-forming steps, we ran simulations with forward flux sampling (FFS), which is used to simulate rare events in nonequilibrium systems with stochastic dynamics^36^ (refer to Supplementary Note 2 for an in-depth explanation). The transition rates for the *i*th edge-forming step were defined as


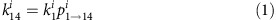


where 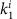 is the rate of forming the first correct bp and 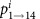 is the probability for this initial bp to lead to the formation of full-length, 14-bp edges. The value of 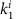 contains information about the diffusive search of complementary modules up to the point when the first bp is formed and is mainly determined by the spatial proximity and orientational alignment of complementary single-stranded segments. On the other hand, 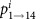 contains information regarding both the propensity of the modules to dissociate after the formation of the first contact and the ability to extend pairing from partial to a complete connection as well. The probability 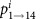 depends both on the stability of each segment and kinetic effects that may hinder the formation of a full-length duplex.

**Figure 2:**
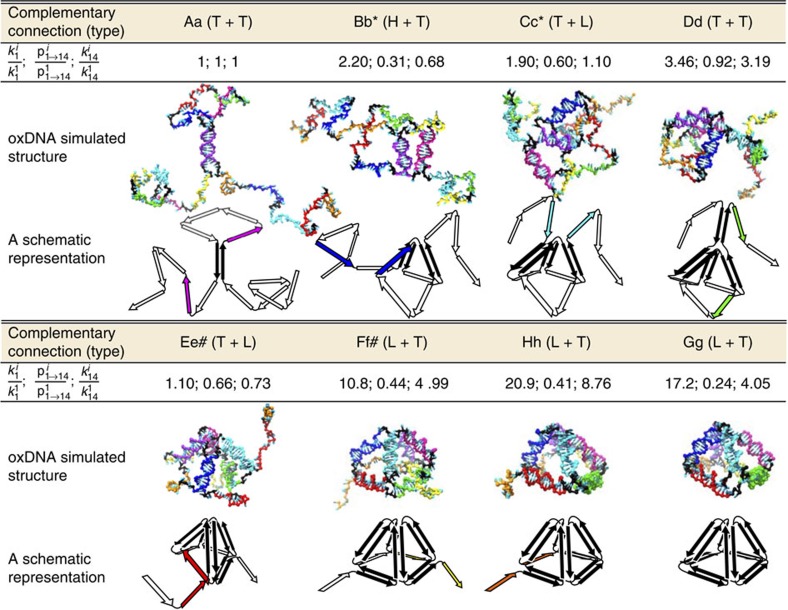
FFS simulations of a designed P1 folding pathway using the oxDNA model. For each connection-forming step, we report three measured quantities, a representative simulation structure and its schematic representation. 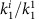 is the relative rate of formation of the first base pair in the sampled *i*th connection. The term 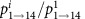 denotes the probability that the formation of the first base pair leads to the successful completion of the *i*th edge relative to that of the first edge. Multiplication of these two quantities yields the relative rate of forming the *i*th connection 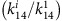. Domains that require the threading of longer tails through a loop are marked with an asterisk (*), whereas the domains that require the threading of shorter tails are marked with a hashtag (#). Black arrows indicate properly formed connections. Coloured arrows indicate the upcoming connection-forming step in the designed folding pathway. It is noteworthy that although a single pathway is considered, owing to the similar thermodynamic stabilities of the Aa and Bb, and Gg and Hh module pairs, pathways where their order of formation is reversed are also likely to play a significant role. Importantly, these alternative pathways also comply with the free-end rules.

**Figure 3:**
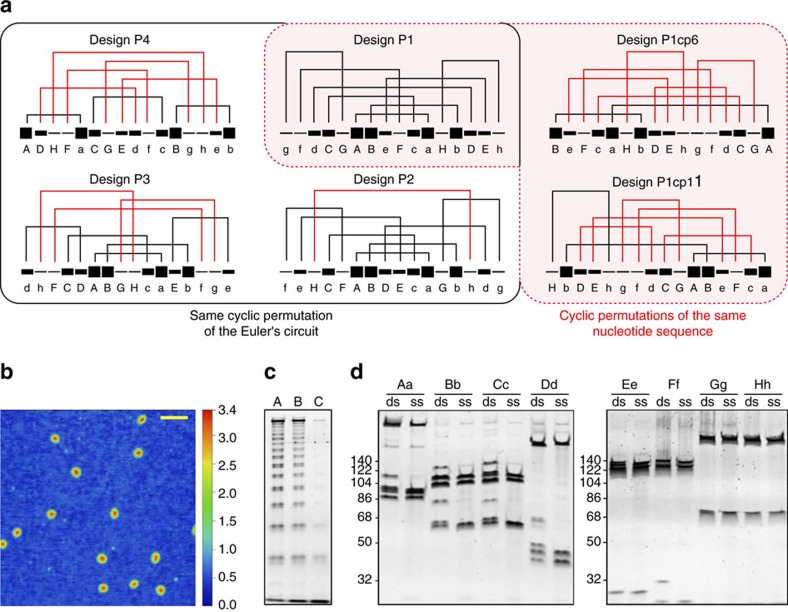
Single-chain DNA square pyramid designs and the structural characterization of P1. (**a**) DNA square pyramid designs were constructed either by rearranging the modules within a prescribed linear topology (black box) or by a circular permutation of the polynucleotide sequence of P1 (red-shaded area). Each line segment depicts one module and is identified with a letter below. Line thickness graphically reflects the stability of complementary module pairs, the order of which was estimated based on the hPin melting curves (Supplementary Fig. 2). The stabilities are depicted on a scale from 1 to 10 and were calculated according to equation (2). Designed folding pathways (a series of connection-forming steps) are depicted for each 4 Py. Connections drawn in red correspond to topologically or kinetically frustrated steps violating the ‘free-end' rule. (**b**) A topography image of P1 particles deposited on mica. Scale bar, 100 nm. (**c**) Denaturing gradient PAGE after the Mung bean nuclease digestion of the slowly temperature annealed P1. Lines 1, 2 and 3 depict samples after 10, 100 and 300 min of digestion, respectively. (**d**) The denaturing gradient PAGE of P1 digestion using distinct endonucleases. Fragments produced from the digestion of end-trimmed double-stranded P1 (ds) served as controls for fragments produced after the digestion of the folded single-chain P1 (ss). Contributions from the antisense (−) strand digestion present in the control samples yet absent from the single-chain P1 containing samples result in additional bands in ds lanes. Experiments were repeated at least twice with comparable results.

Initially, we designed the folding to proceed from the centre of the chain towards the 3′-terminus (Aa→Dd), followed by the threading of the remaining unbound 5′-terminus through the partially folded pyramid (P0; Supplementary Fig. 3a). However, simulations revealed a kinetic bottleneck (observe the small relative rate of forming the 5th connection in Supplementary Fig. 4) caused by the difficulty of threading a long tail through a small loop, which we believe was the most probable cause for experimentally observed lower efficiencies of folding P0 (Supplementary Fig. 3b,c). The subsequently optimized P1 design (Fig. 2) implemented an ‘inside-out' folding strategy in which the longer tails were threaded earlier in the folding process. In addition, the lengths of the free 3′- and 5′-termini were minimized by designing the folding pathway symmetrically around the middle of the chain (refer to P1 in Fig. 3a). The corresponding FFS simulations demonstrated that 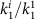 generally increased as the folding progressed, demonstrating cooperativity as a result of the progressively preorganized structure^37^.

### Structural characterization of the P1 single-chain pyramid

A single-stranded polynucleotide P1 chain (286 nt) designed according to the ‘free-end' rule was produced and slowly temperature annealed as described in the Methods section. Native polyacrylamide gel electrophoresis (PAGE) analysis of the slowly annealed P1 single-stranded DNA (ssDNA) revealed a homogeneous band, even at high concentrations (Supplementary Fig. 5a). Atomic force microscopy analysis revealed a homogeneous population of particles with an average height of 2.6±0.1 nm (Fig. 3b and Supplementary Fig. 5b), which is in good agreement with the molecular model (Supplementary Note 2). For comparison, a multi-strand DNA square pyramid was assembled from five oligonucleotides (Supplementary Fig. 6a) in a manner similar to the previously reported multi-chain tetrahedron^38^, which demonstrated comparable hydrodynamic size and shape as the single-stranded design (Supplementary Fig. 6b). Digestion analysis of assembly of P1 using Mung bean nuclease (MBN) (Fig. 3c and Supplementary Fig. 7) and restriction endonucleases (RE) specifically targeting the corresponding edges (Fig. 3d and Supplementary Fig. 8) revealed the pattern of digestion products in agreement with the designed topology.

### Single-chain pyramids violating the free-end rule

To establish the robustness of the design principles, five additional polynucleotide sequences based on the same Eulerian circuit (Supplementary Fig. 1b) were designed. All designs were constructed from the same set of complementary modules (Aa... Hh). To rule out any topological effects on the folding outcomes, P1–P4 were designed from the same cyclic permutation of the Eulerian circuit (Supplementary Fig. 1c). The arrangements of the modules in P2–P4 were designed to encode for folding pathways that included one, three or five steps that violate the ‘free-end' rule, respectively (Fig. 3a), while maintaining the same topology and global thermodynamic stability of the pyramid. To demonstrate that folding is not dictated by distinct cross-vertex sequences, we also designed two circular permutations of the P1 sequence shifted by 6 and 11 modules, respectively, which resulted in 6 and 5 violations of the ‘free-end rule' (P1cp6 and P1cp11 in Fig. 3a). oxDNA FFS simulations of P4 revealed that after the formation of steps Aa→Cc in P4, which form without violating the ‘free-end' rule, the rates of formation dramatically dropped by over four orders of magnitude in the case of Dd (Supplementary Fig. 9), corroborating the qualitative topological constraints.

### Comparing the folding characteristics of DNA pyramids

**Figure 4:**
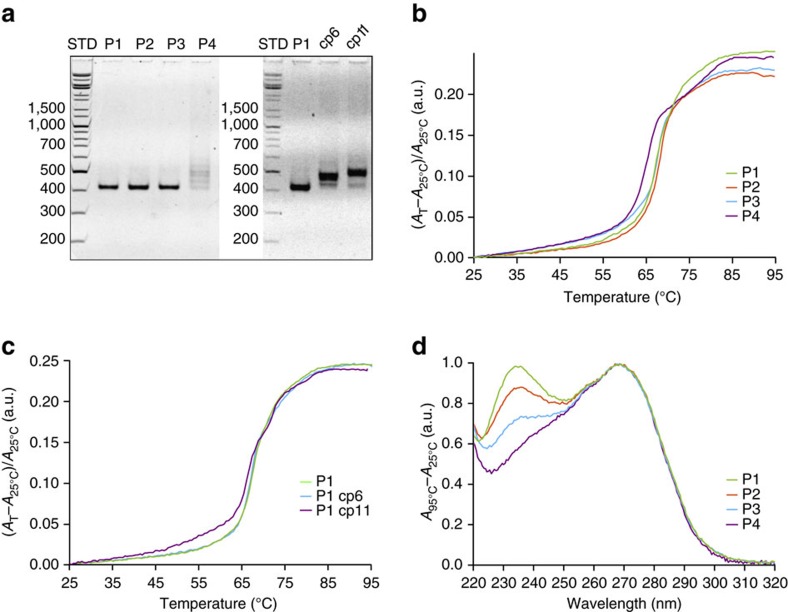
Comparing the properties of slowly annealed square pyramid designs. (**a**) Native PAGE of slowly annealed (rate of annealing: −1 °C min^−1^) P1–P4 (left), and P1 and its circular permutation (cp6 and cp11) designs (right). (**b**) Annealing curves of the P1–P4 designs obtained by measuring the absorbance at 260 nm (annealing rate: −0.2 °C min^−1^). (**c**) Comparison of the P1, P1cp6 and P1cp11 annealing curves measured at 260 nm (−0.2 °C min^−1^). (**d**) Normalized thermal difference spectra of P1–P4. Experiments were repeated at least twice with comparable results.

PAGE analysis of slowly annealed 4 Py designs (Fig. 4a) displayed a single homogeneous band for P1–P3, whereas the annealed structures of P4, and P1cp6 and P1cp11 were heterogeneous, demonstrating misfolded products due to a higher number of topologically unfavourable steps. The ultraviolet absorbance annealing curves of P1–P3 displayed a single major transition, whereas two transitions were detected in the case of annealing of P4 (Fig. 4b). However, lower hyperchromicity was measured in the case of annealing of P2 and P3, indicating a lower fraction of successfully formed base pairs compared with P1 and P4. Contrary to P4, no hysteresis between the annealing and the melting curve of P1 was observed (Supplementary Fig. 10a,b, respectively). Although the P1cp11 displayed multiple transitions, the annealing curve of P1cp6 almost completely overlapped with that of P1 (Fig. 4c). In addition, thermal difference spectra that serve as a spectroscopic signature of nucleic acid structures^39^ revealed clear differences observed between P1 and P4, demonstrating distinct structural features for the different designs (Fig. 4d), where the peak intensity at 236 nm correlated with the number of topologically frustrated steps.

**Figure 5:**
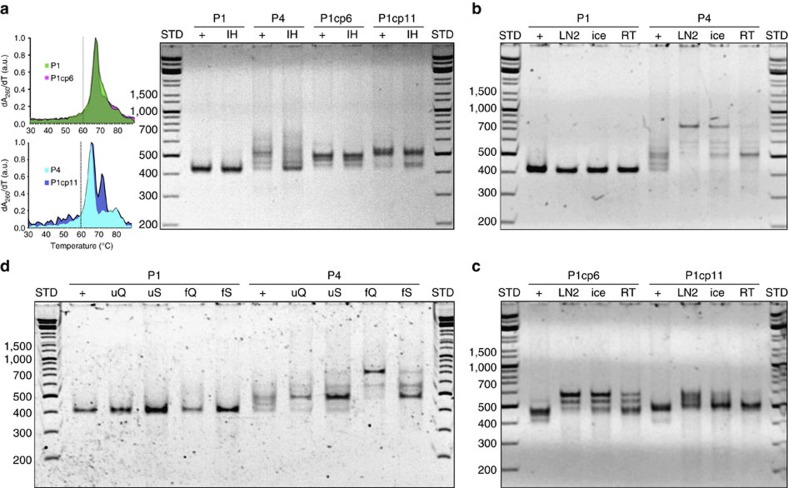
The rapid folding of a single-chain DNA square pyramid demonstrates the feasibility of implemented design principles. (**a**–**d**) All reference samples (+) were annealed slowly (−1 °C min^−1^) at 50 nM in 1 × TAE/Mg^2+^. (**a**) Samples were annealed slowly with an additional 12 h isothermal hold (IH) at 60 °C. Left: dashed lines indicate the position of IH relative to the ‘rate-of-folding' data of designs annealed at −0.2 °C min^−1^ and a concentration of 50 nM in 1 × TAE/Mg^2+^ (measured at 260 nm). Right: an increase in the intensity of the band with mobility comparable to P1 was observed in the case of P4 and P1cp11. P1cp6 required an IH at higher temperatures (65 °C; Supplementary Fig. 10). (**b**) Rapid temperature quenching of P1 and P4 with liquid nitrogen (LN_2_), ice or by transfer to RT. Contrary to P4, the folding of P1 is independent of the annealing protocol. (**c**) P1cp6 and P1cp11 result in distinct patterns depending on the temperature quenching. (**d**) The isothermal annealing of P1 and P4 from 8 M urea (u) or 70% formamide (f) in 1 × TAE/Mg^2+^. Samples were folded quickly (Q) with rapid dilution to 10 × of the initial volume or slowly (S) via dialysis against the 1 × TAE/Mg^2+^ supplemented with 0.8 M urea (or 7% formamide), to ensure equivalent end conditions. Experiments were repeated at least twice with comparable results.

To further analyse the ability of P4, P1cp6 and P1cp11 to fold into the same target fold, we performed slow temperature annealing with an additional 12 h isothermal hold at the lower-temperature boundary of the major transition (Fig. 5a), which has been previously proposed to facilitate the annealing of DNA multi-strand designs^40^. This resulted in the increased intensity of the band with mobility comparable to P1 in the case of P4 and P1cp11 held at 60 °C (Fig. 5a), as well as for P1cp6, which was held at 65 °C (Supplementary Fig. 11). These results imply that prolonged annealing may partially counterbalance the unfavourability of the folding pathway by facilitating escape from kinetic bottlenecks by backtracking.

### Folding by temperature quenching and rapid dilution

Finally, the largest differences in the folding properties of knotted 4 Py designs were expected to be observed under stringent folding conditions, such as temperature quenching or folding by rapid dilution from a chemical denaturant. Temperature quenching was achieved by transfer to room temperature (RT), ice or even by plunging into the liquid nitrogen (Fig. 5b,c). Results demonstrate the robustness of the design of the folding pathway of highly knotted P1, as no differences were observed between the slowly annealed control and rapidly temperature-quenched samples. In contrast, P4, P1cp6 and P1cp11 displayed heterogeneous products that contained very low amounts of the desired product. In addition, the folding efficiency of the multi-strand design (multi-strand DNA square pyramid in Supplementary Fig. 6c) decreased drastically on temperature quenching, demonstrating the merits of using single-chain over multi-chain designs for rapid folding. However, steep temperature gradients remain in the quenching experiments. To evaluate the ability of P1 to assemble isothermally as a prerequisite for folding under physiological conditions, rapid dilution of the chemically unfolded sample (8 M urea or 70% formamide; Fig. 5d) was performed at RT and compared with the control samples prepared via slow dialysis. Regardless of the protocol used, PAGE revealed a single band with the same mobility as that of the thermally annealed control for the P1 design, in contrast to a distinct polydisperse pattern observed with P4.

## Discussion

The successful folding of designed single-chain DNA into a highly knotted target implies that the predicted folding pathways make a dominant contribution to the actual ensemble of folding pathways, and that differences in the sequential arrangements of modules with respect to stability can be used to guide the folding pathway. Although an artificial single-chain RNA nanostructure was recently reported to fold isothermally during enzymatic production^41^, it implemented a non-knotted design based on kissing loop interactions. To our knowledge, P1 is the first and most highly knotted single-chain design that is able to self-assemble isothermally without breaking or forming covalent bonds. A single-chain-designed tetrahedral protein fold was constructed from orthogonal, dimerizing coiled-coil modules^42^, implementing similar principles as DNA nanostructures, which opens the prospects of extending the technology to designed knotted protein structures. We anticipate that the principles outlined in this report will allow for the formation of knotted nanostructures and materials with advanced properties.

## Methods

### Design of modules

To prepare a working set of complementary modules, we generated a pool of dissimilar 14-mer candidate sequences using DNASequenceGenerator v2.01 (ref. 43). Each module was designed using a specific sequence mask ‘snnn**nnnnnn**nnns', where the sequence in bold represents a distinct RE site. Complementary modules were designed according to the criteria of orthogonality and predicted thermal stability, which were evaluated *in silico* using the CANADA software package^44^. To experimentally assess the stability of modules, we designed a set of hairpin-forming (hPin) oligonucleotides (Supplementary Table 1).

### Amplification and purification of ssDNA

All DNA concentrations in solution were determined from measuring A_260_ on a NanoDrop1000 and calculated using theoretically predicted extinction coefficients^45^. Synthetic genes were obtained from GeneWiz in a pUC57 lyophilized vector format. To facilitate PCR amplification of constructs using a common set of primers, sequences were equipped with custom upstream and downstream flanking regions that served as universal flanking sites (Supplementary Table 2). Constructs were amplified using an unmodified forward primer and a 5′-dual biotin HPLC-purified reverse primer (Supplementary Table 3). All desalting steps were performed using Thermo Scientific's GeneJET PCR purification kit. To isolate non-biotinylated sense (+) strand, the streptavidin-coated magnetic beads (Pierce) were conditioned by three consecutive washing steps with 2 × B/W buffer (10 mM Tris-HCl pH 7.5, 1 mM EDTA, 2 M NaCl). Biotinylated PCR amplicons in 1 × B/W buffer were immobilized on streptavidin-coated magnetic beads in a 30-min incubation step at RT. The sense (+) strand was released by alkali denaturation (30 min at RT) in Elution solution (60 mM NaOH, 150 mM NaCl) followed by neutralization/pH stabilization with 500 mM HCl and 10 × TAE/Mg^2+^ (400 mM Tris-HCl, 200 mM acetic acid, 20 mM EDTA, 125 mM MgCl_2_ pH 8.0). Residual biotinylated double-stranded DNA contaminants released from streptavidin during alkali denaturation were separated from pre-annealed ssDNA by 2% AGE. Bands corresponding to ssDNA were excised for DNA extraction with electroelution.

### Removal of universal flanking sequences by end trimming

AgeI and SphI were purchased from NEB at a concentration of 10 U μl^−1^. We first annealed the isolated 4 Py-containing ssDNA at 500 nM in Milli-Q water supplemented with 14 mM MgCl_2_ and 7.5 μM ‘end-trimming' primers (Supplementary Table 4), to generate upstream/downstream double-stranded DNA restriction sites. After annealing, the solution was added 5 × NEBuffer2.1 without magnesium, and restriction enzymes AgeI and SphI (1.2 U pmol^−1^ ssDNA each). The reaction mix was incubated for 5 h at 37 °C. Excess primers and flanking sequences were removed by desalting. We verified the quality of end-trimmed sequences using 8% TBE-polyacrylamide denaturing gels with 40% formamide.

### DNA staining

All polyacrylamide gels used in this study were stained with 1 × SybrGOLD solution for 15–30 min, while shaking gently.

### Annealing of nanostructures

Slow temperature annealing was typically performed in 1 × TAE/Mg^2+^ buffer implementing a quick denaturation step (3 min at 95 °C) followed by gradual cooling from 95 °C to 15 °C at a rate of −1 °C min^−1^, unless stated otherwise.

### Temperature quenching

Before temperature-quenching experiments, we annealed 100 μl stock solutions of respective 50-nM 4-Py samples using slow temperature annealing, to disrupt potentially preformed aggregates and homogenize the solution. In case of annealing the multi-strand pyramid design, five oligonucleotides (Supplementary Table 5) were mixed in equimolar 50 nM concentrations and annealed using slow temperature annealing. Following the annealing, samples were divided into four aliquots, containing 10 μl each, to facilitate thermal equilibration during quenching. Three aliquots were additionally denatured for 5 min at 95 °C and quickly transferred to either liquid nitrogen (30–60 s), on ice (∼10 min) or were allowed to cool down to RT over a period of 30 min. The non-denatured aliquot was used as a reference sample (+). After quenching, samples were thawed and/or held on ice until PAGE. Gel electrophoresis was performed at 25 °C on 6% TBE–polyacrylamide gels.

### Isothermal folding by rapid dilution

Isothermal folding of P1 and P4 was performed either with rapid dilution or dialysis. To perform refolding with rapid dilution, we prepared 12 μl of 125 nM ssDNA in 1 × TAE/Mg^2+^ supplemented either with 8 M urea or 70% formamide. We then quickly added 108 μl of 1.11 × TAE/Mg^2+^ and mixed, to dilute the solution tenfold. All steps were carried out at RT and no prior thermal annealing was carried out on the ssDNA stock solutions. Likewise, to compare the folding outcomes, we performed slow folding by dialysis. For this purpose, we mixed 120 μl of 12.5 nM ssDNA in 1 × TAE/Mg^2+^, supplemented either with 8 M urea or 70% formamide, and dialysed against 1 × TAE/Mg^2+^ with 0.8 M urea or 7% formamide, respectively, to ensure comparable end conditions. Dialysis was performed against 250 ml of buffer at RT for 3 h.

### Structural characterization by enzymatic digestion

Mung Bean Nuclease was purchased from NEB at a concentration of 10 U μl^−1^. For digestion purposes, we annealed 50 μl of 200 nM P1 in Milli-Q water supplemented with 12.5 mM MgCl_2_. All subsequent handling of samples was performed on ice. Samples were divided into aliquots containing 15 μl of solution. Each aliquot was added 1.83 μl of 10 × MBN buffer and 15 U of MBN followed by the immediate transfer to a thermal cycler preheated to 30 °C for a given time period. At specified time intervals, samples were frozen with liquid nitrogen and stored at −20 °C until the end of the experiment. Denaturing PAGE was performed at 45 °C with 10–20% gradient TBE–polyacrylamide gels supplemented with 40% formamide. Samples were added 2 × *V*_sample_ of Denaturing solution (95% formamide, 5 mM EDTA) and were heated to 95 °C for 5 min immediately before loading, as the increased incubation times in denaturing solution resulted in spoilage.

SalI, SmaI, EcoRV, BamHI, EcoRI, HindIII, BsrGI and SpeI were purchased from NEB at a concentration of 10 U μl^−1^. Before restriction analysis, we annealed single-chain P1 (4 pmol at 133 nM) in Milli-Q water supplemented with 14.2 mM Mg^2+^ (MgCl_2_ or MgOAc; depending on RE requirements). After annealing, samples were added appropriate 5 × NEBuffer without Mg^2+^ and 40 U of restriction enzyme. Samples were incubated at 37 °C for 1.5 h, followed by a 20-min inactivation step at 65 °C. Subsequent sample preparation steps and denaturing PAGE analysis was performed as described for MBN digestion. Refer to Supplementary Table 6 for a list of expected fragment lengths resulting from RE digestion of P1.

### Atomic force microscopy

Measurements were performed on an Agilent 5500 in tapping mode using Arrow PointProbe silicon-nitride cantilevers with a nominal tip curvature radius below 10 nm. Ten microlitres of annealed 100 nM pyramid sample in 1 × TAE/Mg^2+^ buffer was deposited on a freshly cleaved mica and incubated for 30 min. Samples were dried under a stream of pure nitrogen gas before imaging without rinsing.

### Thermodynamic analysis

Ultraviolet spectra and annealing/melting curves of 4 Pys and hPins were measured on a Varian Carry 100 Bio ultraviolet–visible spectrophotometer in Buffer A (10 mM Tris-HCl pH 8, 5 mM acetic acid, 0.5 mM EDTA and 12.5 mM MgCl_2_) at 500 nM and 5 μM concentrations, respectively. Thermal difference spectra were obtained by subtracting buffer background-corrected (background measured at 350 nm) ultraviolet spectra obtained at 25 °C from spectra recorded at 95 °C and normalizing the values from 0 to 1. Evaporation was prevented by overlaying with mineral oil (Sigma) and tightly sealing the cuvettes using caps wrapped with Teflon tape.

### Molecular simulation of pyramids

The oxDNA model is a coarse-grained model of DNA at the nucleotide level. The potential energy of a particular configuration includes the following interactions: stacking, cross-stacking, coaxial stacking, hydrogen bonding, excluded volume and backbone chain connectivity. Base-pairing interactions obey Watson–Crick specificity (that is, A-T or G-C pairs), but other interactions such as Hoogsteen pairs are excluded from the model. Details of the interactions contributing to the oxDNA potential can be found elsewhere^34^ and the simulation code for oxDNA can be downloaded from the oxDNA website (https://dna.physics.ox.ac.uk). Here, the model is used to calculate transition rates of module formation in the P0, P1 and P4 pyramid designs. Kinetic results are obtained by performing molecular dynamics simulations using an Anderson-like thermostat^46^. Formation rates of modules are computed using FFS^35^. A detailed description of the numerical methods is presented in Supplementary Note 2. Data obtained from the FFS simulations are listed in Supplementary Tables 8–10.

### Calculation of line thickness

Experimentally determined thermal stabilities of hPins were used to calculate the thickness of lines in Fig. 3a according to


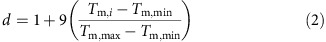


*T*_m,min_ and *T*_m, max_ denote melting temperatures of the least (*F*–*f*) and the most (*A*–*a*) stable hPin, respectively.

## Additional information

**How to cite this article:** Kočar, V. *et al*. Design principles for rapid folding of knotted DNA nanostructures. *Nat. Commun.* 7:10803 doi: 10.1038/ncomms10803 (2016).

## Supplementary Material

Supplementary Information Supplementary Figures 1-13, Supplementary Tables 1-10, Supplementary Notes 1-2 and Supplementary References

## References

[b1] Anfinsen C. B. The formation and stabilization of protein structure. Biochem. J. 128, 737–749 (1972) . 4565129 10.1042/bj1280737 PMC1173893

[b2] Drexler K. E. Molecular engineering: an approach to the development of general capabilities for molecular manipulation. Proc. Natl Acad. Sci. USA 78, 5275–5278 (1981) . 16593078 10.1073/pnas.78.9.5275 PMC348724

[b3] Dirks R. M., Lin M., Winfree E. & Pierce N. A. Paradigms for computational nucleic acid design. Nucleic Acids Res. 32, 1392–1403 (2004) . 14990744 10.1093/nar/gkh291 PMC390280

[b4] Forgan R. S., Sauvage J. P. & Stoddart J. F. Chemical topology: complex molecular knots, links, and entanglements. Chem. Rev. 111, 5434–5464 (2011) . 21692460 10.1021/cr200034u

[b5] Sulkowska J. I., Rawdon E. J., Millett K. C., Onuchic J. N. & Stasiak A. Conservation of complex knotting and slipknotting patterns in proteins. Proc. Natl Acad. Sci. USA 109, E1715–E1723 (2012) . 22685208 10.1073/pnas.1205918109 PMC3387036

[b6] Schmidberger J. W., Wilce J. A., Weightman A. J., Whisstock J. C. & Wilce M. C. The crystal structure of DehI reveals a new alpha-haloacid dehalogenase fold and active-site mechanism. J. Mol. Biol. 378, 284–294 (2008) . 18353360 10.1016/j.jmb.2008.02.035

[b7] Bolinger D. . A Stevedore's protein knot. PLoS Comput. Biol. 6, e1000731 (2010) . 20369018 10.1371/journal.pcbi.1000731 PMC2848546

[b8] Sayre T. C., Lee T. M., King N. P. & Yeates T. O. Protein stabilization in a highly knotted protein polymer. Protein Eng. Des. Sel. 24, 627–630 (2011) . 21669955 10.1093/protein/gzr024 PMC3165941

[b9] Sulkowska J. I., Sulkowski P., Szymczak P. & Cieplak M. Stabilizing effect of knots on proteins. Proc. Natl Acad. Sci. USA 105, 19714–19719 (2008) . 19064918 10.1073/pnas.0805468105 PMC2604914

[b10] King N. P., Jacobitz A. W., Sawaya M. R., Goldschmidt L. & Yeates T. O. Structure and folding of a designed knotted protein. Proc. Natl Acad. Sci. USA 107, 20732–20737 (2010) . 21068371 10.1073/pnas.1007602107 PMC2996448

[b11] Seeman N. C. DNA in a material world. Nature 421, 427–431 (2003) . 12540916 10.1038/nature01406

[b12] Seeman N. C. Nanomaterials based on DNA. Annu. Rev. Biochem. 79, 65–87 (2010) . 20222824 10.1146/annurev-biochem-060308-102244 PMC3454582

[b13] Pinheiro A. V., Han D., Shih W. M. & Yan H. Challenges and opportunities for structural DNA nanotechnology. Nat. Nanotechnol. 6, 763–772 (2011) . 22056726 10.1038/nnano.2011.187 PMC3334823

[b14] Chen J. H. & Seeman N. C. Synthesis from DNA of a molecule with the connectivity of a cube. Nature 350, 631–633 (1991) . 2017259 10.1038/350631a0

[b15] Zhang Y. & Seeman N. Construction of a DNA-truncated octahedron. J. Am. Chem. Soc. 116, 1661–1669 (1994) .

[b16] Winfree E., Liu F., Wenzler L. A. & Seeman N. C. Design and self-assembly of two-dimensional DNA crystals. Nature 394, 539–544 (1998) . 9707114 10.1038/28998

[b17] Yan H., LaBean T. H., Feng L. & Reif J. H. Directed nucleation assembly of DNA tile complexes for barcode-patterned lattices. Proc. Natl Acad. Sci. USA 100, 8103–8108 (2003) . 12821776 10.1073/pnas.1032954100 PMC166189

[b18] Rothemund P. W. Folding DNA to create nanoscale shapes and patterns. Nature 440, 297–302 (2006) . 16541064 10.1038/nature04586

[b19] Shih W., Quispe J. & Joyce G. A 1.7-kilobase single-stranded DNA that folds into a nanoscale octahedron. Nature 427, 618–621 (2004) . 14961116 10.1038/nature02307

[b20] Andersen E. S. . Self-assembly of a nanoscale DNA box with a controllable lid. Nature 459, 73–76 (2009) . 19424153 10.1038/nature07971

[b21] Douglas S. M. . Rapid prototyping of 3D DNA-origami shapes with caDNAno. Nucleic Acids Res. 37, 5001–5006 (2009) . 19531737 10.1093/nar/gkp436 PMC2731887

[b22] Ke Y., Ong L. L., Shih W. M. & Yin P. Three-dimensional structures self-assembled from DNA bricks. Science 338, 1177–1183 (2012) . 23197527 10.1126/science.1227268 PMC3843647

[b23] Wei B., Dai M. & Yin P. Complex shapes self-assembled from single-stranded DNA tiles. Nature 485, 623–626 (2012) . 22660323 10.1038/nature11075 PMC4238960

[b24] Li Z. . A replicable tetrahedral nanostructure self-assembled from a single DNA strand. J. Am. Chem. Soc. 131, 13093–13098 (2009) . 19737020 10.1021/ja903768f PMC3083857

[b25] He X., Dong L., Wang W., Lin N. & Mi Y. Folding single-stranded DNA to form the smallest 3D DNA triangular prism. Chem. Commun. 49, 2906–2908 (2013) . 10.1039/c3cc39266j 23459629

[b26] Seeman N. C. Construction of three-dimensional stick figures from branched DNA. DNA Cell Biol. 10, 475–486 (1991) . 1892564 10.1089/dna.1991.10.475

[b27] Bucka A. & Stasiak A. Construction and electrophoretic migration of single-stranded DNA knots and catenanes. Nucleic Acids Res. 30, e24 (2002) . 11884643 10.1093/nar/30.6.e24 PMC101368

[b28] Mashaghi A., van Wijk R. J. & Tans S. J. Circuit topology of proteins and nucleic acids. Structure 22, 1227–1237 (2014) . 25126961 10.1016/j.str.2014.06.015

[b29] Romano F., Hudson A., Doye J. P. K., Ouldridge T. E. & Louis A. A. The effect of topology on the structure and free energy landscape of DNA kissing complexes. J. Chem. Phys. 136, 215102 (2012) . 22697570 10.1063/1.4722203

[b30] Turberfield A. J. . DNA fuel for free-running nanomachines. Phys. Rev. Lett. 90, 118102 (2003) . 12688969 10.1103/PhysRevLett.90.118102

[b31] Klavžar S. & Rus J. Stable traces as a model for self-assembly of polypeptide nanoscale polyhedrons. MATCH Commun. Math. Comput. Chem. 70, 317–330 (2013) .

[b32] Fijavž G., Pisanski T. & Rus J. Strong traces model of self-assembly polypeptide structures. MATCH Commun. Math. Comput. Chem. 71, 199–212 (2014) .

[b33] Lua R. C. PyKnot: a PyMOL tool for the discovery and analysis of knots in proteins. Bioinformatics 28, 2069–2071 (2012) . 22611132 10.1093/bioinformatics/bts299

[b34] Ouldridge T. E., Louis A. A. & Doye J. P. Structural, mechanical, and thermodynamic properties of a coarse-grained DNA model. J. Chem. Phys. 134, 85101 (2011) . 10.1063/1.3552946 21361556

[b35] Doye J. P. K. . Coarse-graining DNA for simulations of DNA nanotechnology. Phys. Chem. Chem. Phys. 15, 20395–20414 (2013) . 24121860 10.1039/c3cp53545b

[b36] Allen R. J., Valeriani C. & ten Wolde P. R. Forward flux sampling for rare event simulations. J. Phys. Condens. Matter 21, 463102 (2009) . 21715864 10.1088/0953-8984/21/46/463102

[b37] Mugler A., Tans S. J. & Mashaghi A. Circuit topology of self-interacting chains: implications for folding and unfolding dynamics. Phys. Chem. Chem. Phys. 16, 22537–22544 (2014) . 25228051 10.1039/c4cp03402c

[b38] Goodman R. P. . Rapid chiral assembly of rigid DNA building blocks for molecular nanofabrication. Science 310, 1661–1665 (2005) . 16339440 10.1126/science.1120367

[b39] Mergny J. L., Li J., Lacroix L., Amrane S. & Chaires J. B. Thermal difference spectra: a specific signature for nucleic acid structures. Nucleic Acids Res. 33, e138 (2005) . 16157860 10.1093/nar/gni134 PMC1201377

[b40] Sobczak J. P., Martin T. G., Gerling T. & Dietz H. Rapid folding of DNA into nanoscale shapes at constant temperature. Science 338, 1458–1461 (2012) . 23239734 10.1126/science.1229919

[b41] Geary C., Rothemund P. W. K. & Andersen E. S. A single-stranded architecture for cotranscriptional folding of RNA nanostructures. Science 345, 799–804 (2014) . 25124436 10.1126/science.1253920

[b42] Gradisar H. . Design of a single-chain polypeptide tetrahedron assembled from coiled-coil segments. Nat. Chem. Biol. 9, 362–366 (2013) . 23624438 10.1038/nchembio.1248 PMC3661711

[b43] Feldkamp U., Saghafi S., Banzhaf W. & Rauhe H. in Lecture Notes in Computer Science, Vol. 2340, eds Jonoska N., Seeman N. C. Ch. 3, 23–32Springer (2002) .

[b44] Feldkamp U. CANADA: designing nucleic acid sequences for nanobiotechnology applications. J. Comp. Chem. 31, 660–663 (2010) . 19530109 10.1002/jcc.21353

[b45] Tataurov A. V., You Y. & Owczarzy R. Predicting ultraviolet spectrum of single stranded and double stranded deoxyribonucleic acids. Biophys. Chem. 133, 66–70 (2008) . 18201813 10.1016/j.bpc.2007.12.004

[b46] Sulc P. . Sequence-dependent thermodynamics of a coarse-grained DNA model. J. Chem. Phys. 137, 135101 (2012) . 23039613 10.1063/1.4754132

