# Supplementary material for: Design principles for rapid folding of knotted DNA nanostructures
Source: Nat Commun. 2016 Feb 18;7:10803. doi: 10.1038/ncomms10803 (PMC4759626; doi:10.1038/ncomms10803)
Supplement: Supplementary Information — Supplementary Figures 1-13, Supplementary Tables 1-10, Supplementary Notes 1-2 and Supplementary References [file ncomms10803-s1.pdf]

Supplementary Figures

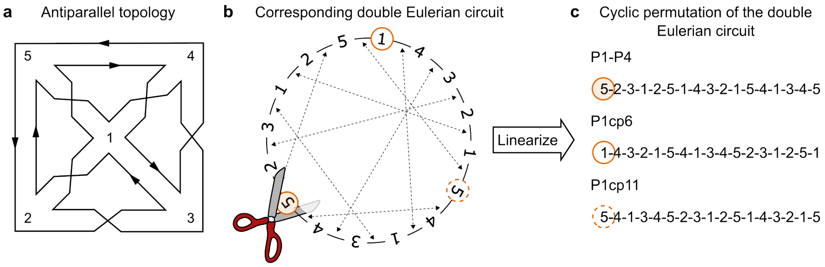

Supplementary Figure 1 | The antiparallel single-chain pyramid topology used for the design of DNA nanostructures, and the corresponding double Eulerian circuit. (**a**) Out of five theoretically available antiparallel topologies a topology that does not cross at the 4-valent vertex (labeled as 1) was chosen. Thus, crossing is topologically required to occur only at the edges. The chain traverses each edge of the pyramid exactly twice and in an antiparallel orientation to comply with the antiparallel nature of DNA. Crossings of the chain are depicted for illustration purposes only, and are not intended to display the actual positions of the crossings that lead to knotting. (**b**) The chain can be traced along the edges of the pyramid using a circular sequence of consecutively traversed vertices, which defines a double Eulerian circuit. (**c**) In order to design a polynucleotide (or an equivalent biopolymer) sequence, a site of linearization must be decided upon, which is achieved by omitting one linker from the circuit. In the case of P1-P4 single-chain pyramid designs, the site of linearization was the same, while in the case of P1cp6 and P1cp11, the site of linearization was shifted by 6 and 11 modules relative to P1's, respectively.

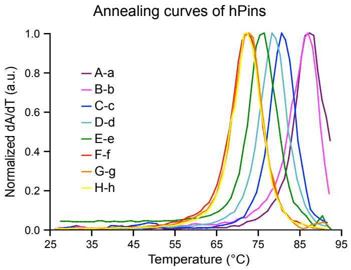

Supplementary Figure 2 | Experimentally determined thermal stabilities of hairpins (hPin). To estimate the order of connection-forming steps for the folding of 4Pys based on the stabilities of modules, we designed hairpin-forming oligonucleotides containing single pairs of complementary modules (refer to Supplementary Table 1 for polynucleotide sequences). Melting curves were obtained as described in the Methods section.

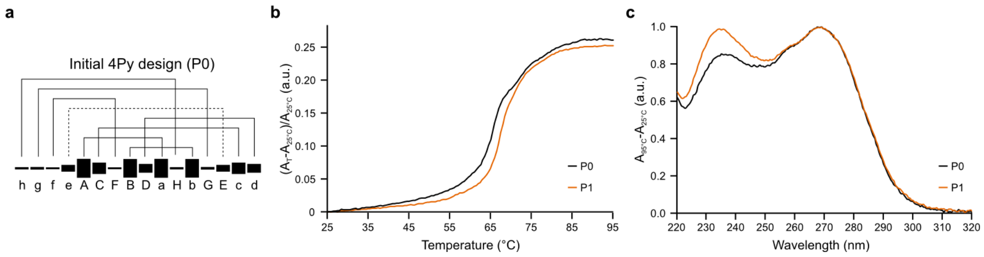

Supplementary Figure 3 I The initial single-chain pyramid design (P0). (**a**) The designed folding order of P0 displays a two-part nature, where the folding initially proceeds from the centre towards the 3'-terminus, followed by the threading of 5'-terminus through the partially formed structure. A dotted connection denotes a kinetically unfavorable step, as discerned from the FFS simulations. A comparison between the respective annealing curves (**b**), and thermal difference spectra (**c**) of P0 and the subsequently improved P1 design is depicted.

Complementary connection (type)	Aa (T + T)	Bb (H + T)	Cc (L + T)	Dd (L + T)
$\frac{k_1^i}{k_1^1}$; $\frac{\mathbf{p^i_{1\to14}}}{\mathbf{p^1_{1\to14}}}$; $\frac{k^i_{14}}{k^1_{14}}$	1; **1**; *1*	1.17; **0.14**; *0.16*	1.38; **0.12**; *0.17*	26.00; **0.92**; *23.92*

oxDNA simulated structure

A schematic representation

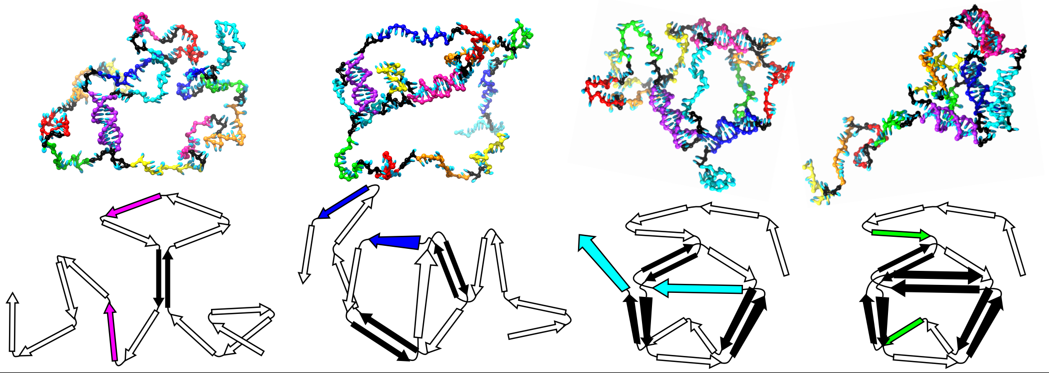

Complementary connection (type)	Ee (L + T)	Ff (L + T)	Gg (L + T)	Hh (L + T)
$\frac{k_1^i}{k_1^1}$; $\frac{\mathbf{p^i_{1\to14}}}{\mathbf{p^1_{1\to14}}}$; $\frac{k^i_{14}}{k^1_{14}}$	0.39; **4x10^{-4};** *2x10^{-4}*	6.93; **0.02**; *0.14*	15.73; **0.01;** *0.16*	21.18; **0.37**; *7.84*

oxDNA simulated structure

A schematic representation

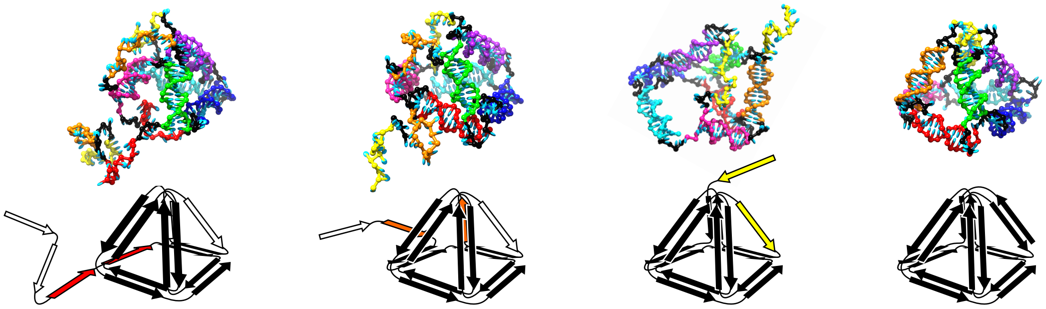

Supplementary Figure 4 | Relative rates and probabilities obtained from FFS simulations of the designed P0 folding pathway. For description of the parameters refer to Fig. 2 in the main text. After the formation of Aa, Bb, Cc, and Dd, the rate of Ee formation, which involved threading a long tail through a small loop, decreased by over four orders of magnitude. As with P1, because of the similar stabilities of the Aa and Bb, and Gg and Hh complementary module pairs, paths where their order of formation is reversed may also be relevant. If Bb forms before Aa, the resulting pathway also obeys the "free-end" rule. By contrast, if Hh were to form before Gg, the final step would then involve an unfavourable "L+L" step. However, simulations indicate that Gg is significantly more likely to form before Hh due to the much closer proximity of the former two modules once connections Aa→Ff have formed already.

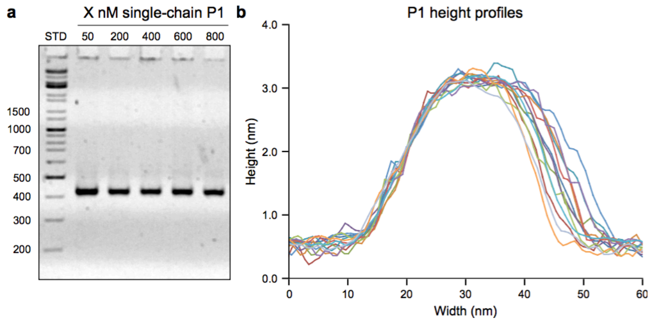

Supplementary Figure 5 I Size characterization of thermally annealed P1 particles. **(a)** Single-chain P1 does not aggregate discernibly, even when folded at up to 800 nM concentrations. (**b**) Height profiles of P1 particles were extracted from the topography images obtained with the atomic force microscopy.

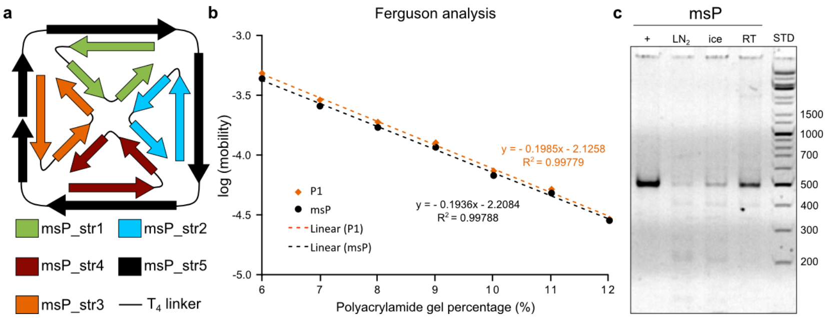

Supplementary Figure 6 I Comparison of P1 to the multi-strand square pyramid (msP). (**a**) A schematic representation of the multi-strand pyramid construction. Refer to Supplementary Table 5 for the actual oligonucleotide sequences. (**b**) Ferguson analysis of P1 and msP displays similar slopes. (**c**) Equimolar samples of msP quenched with liquid nitrogen (LN_2), ice or at room temperature (RT) were directly compared to the thermally annealed control (+).

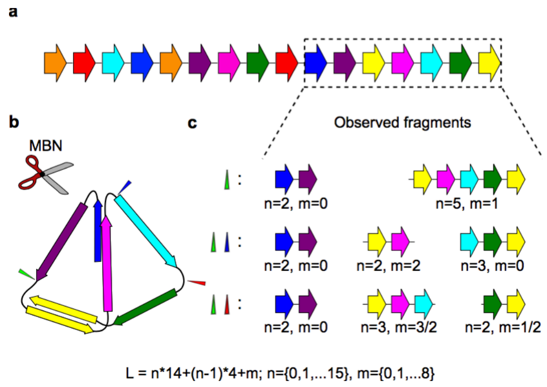

Supplementary Figure 7 I How restrictional analysis of the P1 design using Mung Bean nuclease leads to distinct size classes of bands observed in Fig. 3c. Only the 3'-terminus of the P1 sequence is shown in a schematic 3D representation (**a**) in order to demonstrate why digestion with Mung Bean nuclease (**b**) results in distinct size classes of bands instead of discrete bands as observed on denaturing PAGE (**c**). Each size class observed on denaturing PAGE corresponds to a value of n, i.e., the number of modules in the observed fragments. Length variations within a size class are due to the flanking linkers, which are 4 nt long, being digested to various extents.

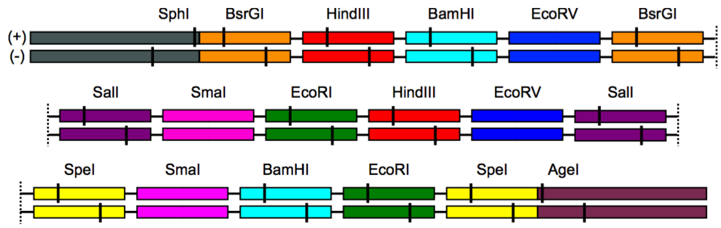

Supplementary Figure 8 I A schematic depiction of the restriction endonuclease cutting profiles depicting the variations in lengths of resulting fragments after (+) sense vs. (-) sense strand digestion. For a complete list of expected fragment lengths after digestion refer to Supplementary Table 6.

Complementary connection (type)	Aa (T + T)	Bb (H + T)	Cc (L + T)	Dd (L + T)
$\frac{k_1^i}{k_1^1}$; $\frac{\mathbf{p}^i_{1\to14}}{\mathbf{p}^1_{1\to14}}$; $\frac{k^i_{14}}{k^1_{14}}$	1; **1**; *1*	0.83; **0.43**; *0.33*	0.39; **0.89**; *0.32*	0.24; **1.6x10^{-4}**; *4.0x10^{-5}*
oxDNA simulated structure	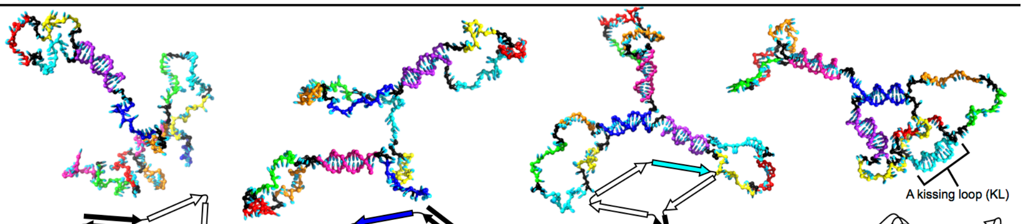			
A schematic representation	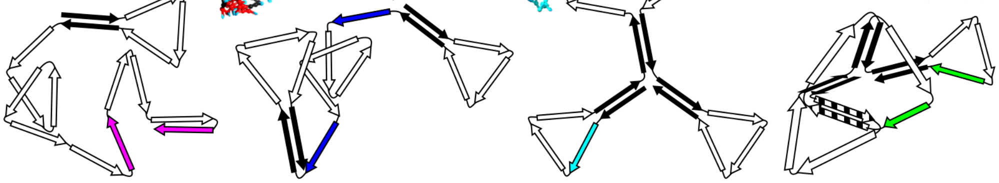			

Supplementary Figure 9 | Relative rates and probabilities obtained from FFS simulations of the designed P4 folding pathway. For description of parameters refer to Fig. 2 in the main text. After the formation of Aa, Bb and Cc, the rate of Dd formation decreased drastically, rendering the sampling of successive domains unattainable. The formation of a kissing loop in the case of Dd is schematically presented with hatched arrows.

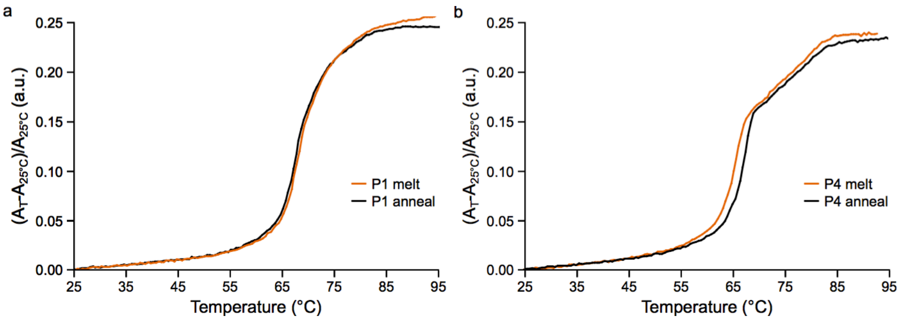

Supplementary Figure 10 | Successive annealing/denaturation curves were measured for P1 (**a**) and P4 (**b**) at 260 nm. A discernible hysteresis was observed in the case of P4 around the major transition.

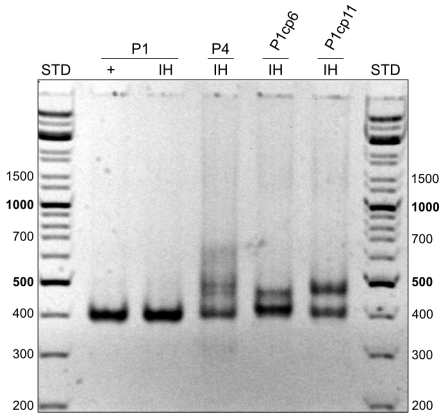

Supplementary Figure 11 I Slow temperature annealing with an isothermal hold at 65°C. The reference sample (+) was annealed slowly (-1°C/min) at 50 nM in 1xTAE/Mg2+. The remaining samples were annealed comparably with an additional 12h isothermal hold (IH) at 65°C. An increase in the intensity of the band with mobility comparable to P1 was observed in all cases.

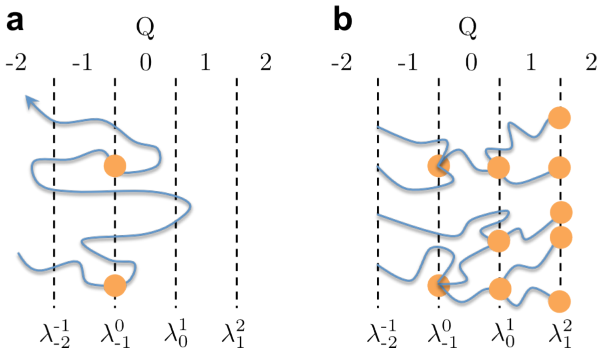

Supplementary Figure 12 I Direct forward flux sampling (FFS). (**a**) A schematic illustration of the interfaces involved in flux generation. The flux is initially measured across the interface λ_{-1}^{0}. The orange dots indicate that a crossing by a trajectory contributes to the flux. These are also the states that are used to launch successive stages of the simulation. (**b**) In direct FFS, large numbers of configurations are randomly selected from the set that successfully crossed the interface λ_{-1}^{0}, and the probability of subsequently crossing the λ_{0}^{1} interface (rather than returning to $Q = -2$) is measured. The process is then iterated over successively chosen interfaces until reaching $Q_{\max}$. Figure adapted from reference reference 1.

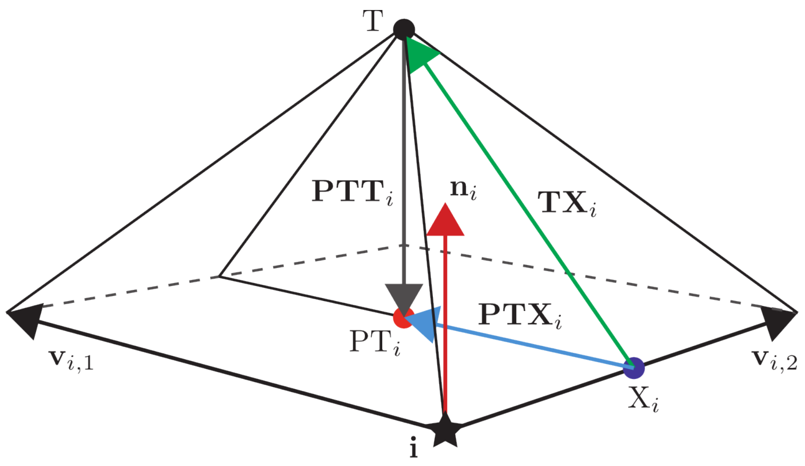

Supplementary Figure 13 | The geometry of the pyramid used to measure its height. Star symbol represents vertex *i* at the base of the pyramid, which is defined by the normal vector $n_i = v_{i,2} \times v_{i,1}$. A black dot indicates the point T, which is the centre of mass of the linkers at the top of the pyramid, and a red dot shows the projection of the point T onto the plane defined by n_i at the point X_i. A purple dot indicates the centre of mass of module *i*. Vectors used in the calculation of the height are also shown.

Supplementary Tables

Supplementary Table 1 I Nucleotide sequences of PAGE-purified hPin oligonucleotides. Reverse complementary modules used for construction are depicted in black, while the T_4 - linkers are coloured in red. Nomenclature of modules is consistent with the nomenclature used for reporting the 4Py's design.

hPin name	Nucleotide sequence
A-a	gcgtgtcgaccgcgttttcgcggtcgacacgc
B-b	gggtcccgggtgtcttttgacacccgggaccc
C-c	gggcgatatcctcgttttcgaggatatcgccc
D-d	ccctggatcctttcttttgaaaggatccaggg
E-e	gggggaattctatcttttgatagaattccccc
F-f	catgaagctttatgttttcataaagcttcatg
G-g	catctgtacatgtgttttcacatgtacagatg
H-h	cagtactagtaacgttttcgttactagtactg

Supplementary Table 2 I Synthetic gene constructs used for PCR amplification of 4Py-containing dsDNA fragments. Polynucleotide sequences of 4Pys are shown in *italics,* with the linkers positioned in vertices are shown in red. Universal flanking sequences used for PCR amplification are underlined and the restriction sites used for "end-trimming" are represented with a bold typeface.

P1 sequence	TCTAGACCTAATACAACTAC**GCATGC**CACATGTACAGATGTTTTCATAAAGCTTCA<br>TGTTTTGAAAGGATCCAGGGTTTTGGGCGATATCCTCGTTTTCATCTGTACATGTG<br>TTTTGCGTGTCGACCGCGTTTTGGGTCCCGGGTGTCTTTTGATAGAATTCCCCCTT<br>TTCATGAAGCTTTATGTTTTCGAGGATATCGCCCTTTTCGCGGTCGACACGCTTTT<br>CAGTACTAGTAACGTTTTGACACCCGGGACCCTTTTCCCTGGATCCTTTCTTTTGG<br>GGGAATTCTATCTTTTCGTTACTAGTACTG**ACCGGT**CTCACACTTTAATCCTGCAG
P2 sequence	TCTAGACCTAATACAACTAC**GCATGC**CATAAAGCTTCATGTTTTGATAGAATTCCC<br>CCTTTTCAGTACTAGTAACGTTTTGGGCGATATCCTCGTTTTCATGAAGCTTTATG<br>TTTTGCGTGTCGACCGCGTTTTGGGTCCCGGGTGTCTTTTCCCTGGATCCTTTCTT<br>TTGGGGGAATTCTATCTTTTCGAGGATATCGCCCTTTTCGCGGTCGACACGCTTTT<br>CATCTGTACATGTGTTTTGACACCCGGGACCCTTTTCGTTACTAGTACTGTTTTGA<br>AAGGATCCAGGGTTTTCACATGTACAGATG**ACCGGT**CTCACACTTTAATCCTGCAG
P3 sequence	TCTAGACCTAATACAACTAC**GCATGC**GAAAGGATCCAGGGTTTTCGTTACTAGTAC<br>TGTTTTCATGAAGCTTTATGTTTTGGGCGATATCCTCGTTTTCCCTGGATCCTTTC<br>TTTTGCGTGTCGACCGCGTTTTGGGTCCCGGGTGTCTTTTCATCTGTACATGTGTT<br>TTCAGTACTAGTAACGTTTTCGAGGATATCGCCCTTTTCGCGGTCGACACGCTTTT<br>GGGGGAATTCTATCTTTTGACACCCGGGACCCTTTTCATAAAGCTTCATGTTTTCA<br>CATGTACAGATGTTTTGATAGAATTCCCCC**ACCGGT**CTCACACTTTAATCCTGCAG
P4 sequence	TCTAGACCTAATACAACTAC**GCATGC**GCGTGTCGACCGCGTTTTCCCTGGATCCTT<br>TCTTTTCAGTACTAGTAACGTTTTCATGAAGCTTTATGTTTTCGCGGTCGACACGC<br>TTTTGGGCGATATCCTCGTTTTCATCTGTACATGTGTTTTGGGGGAATTCTATCTT<br>TTGAAAGGATCCAGGGTTTTCATAAAGCTTCATGTTTTCGAGGATATCGCCCTTTT<br>GGGTCCCGGGTGTCTTTTCACATGTACAGATGTTTTCGTTACTAGTACTGTTTTGA<br>TAGAATTCCCCCTTTTGACACCCGGGACCC**ACCGGT**CTCACACTTTAATCCTGCAG
P1cp6 sequence	TCTAGACCTAATACAACTAC**GCATGC**GGGTCCCGGGTGTCTTTTGATAGAATTCCC<br>CCTTTTCATGAAGCTTTATGTTTTCGAGGATATCGCCCTTTTCGCGGTCGACACGC<br>TTTTCAGTACTAGTAACGTTTTGACACCCGGGACCCTTTTCCCTGGATCCTTTCTT<br>TTGGGGGAATTCTATCTTTTCGTTACTAGTACTGTTTTCACATGTACAGATGTTTT<br>CATAAAGCTTCATGTTTTGAAAGGATCCAGGGTTTTGGGCGATATCCTCGTTTTCA<br>TCTGTACATGTGTTTTGCGTGTCGACCGCG**ACCGGT**CTCACACTTTAATCCTGCAG
P1cp11 sequence	TCTAGACCTAATACAACTAC**GCATGC**CAGTACTAGTAACGTTTTGACACCCGGGAC<br>CCTTTTCCCTGGATCCTTTCTTTTGGGGGAATTCTATCTTTTCGTTACTAGTACTG<br>TTTTCACATGTACAGATGTTTTCATAAAGCTTCATGTTTTGAAAGGATCCAGGGTT<br>TTGGGCGATATCCTCGTTTTCATCTGTACATGTGTTTTGCGTGTCGACCGCGTTTT<br>GGGTCCCGGGTGTCTTTTGATAGAATTCCCCCTTTTCATGAAGCTTTATGTTTTCG<br>AGGATATCGCCCTTTTCGCGGTCGACACGC**ACCGGT**CTCACACTTTAATCCTGCAG

Supplementary Table 3 I Primers used for PCR amplification of 4Py-containing dsDNA fragments. Primers were designed to anneal within the universal flanking regions of synthetic gene constructs. The reverse primer was modified with a dual biotin group necessary to reduce the dissociation rate of the bound antisense (-) strand from the streptavidin coated magnetic beads during the alkaline denaturation step.

F_Lflank1	ACCTAATACAACTACGCATG
5'2-Bio_R_Rflank1	CAGGATTAAAGTGTGAGAC

Supplementary Table 4 l End-trimming oligonucleotides. After performing the alkaline denaturation step we obtain 4Py ssDNA that contains the universal flanking sequences. In order to remove the flanking sequences (we refer to this step as "end-trimming"), we must first generate dsDNA RE sites via thermal annealing (shown in bold) in the presence of end-trimming oligonucleotides before cutting with RE enzymes.

et_Lflank1_SphI_(+)P1	CATGTG**GCATGC**GTAGTTGTATTAG
et_Rflank1_AgeI_(+)P1	ATTAAAGTGTGAG**ACCGGT**CAGTAC
et_Lflank1_SphI_(+)P2	TTTATG**GCATGC**GTAGTTGTATTAG
et_Rflank1_AgeI_(+)P2	ATTAAAGTGTGAG**ACCGGT**CATCTG
et_Lflank1_SphI_(+)P3	CCTTTC**GCATGC**GTAGTTGTATTAG
et_Rflank1_AgeI_(+)P3	ATTAAAGTGTGAG**ACCGGT**GGGGGA
et_Lflank1_SphI_(+)P4	ACACGC**GCATGC**GTAGTTGTATTAG
et_Rflank1_AgeI_(+)P4	ATTAAAGTGTGAG**ACCGGT**GGGTCC
et_Lflank1_SphI_(+)P1cp6	GGACCC**GCATGC**GTAGTTGTATTAG
et_Rflank1_AgeI_(+)P1cp6	ATTAAAGTGTGAG**ACCGGT**CGCGGT
et_Lflank1_SphI_(+)P1cp11	GTACTG**GCATGC**GTAGTTGTATTAG
et_Rflank1_AgeI_(+)P1cp11	ATTAAAGTGTGAG**ACCGGT**GCGTGT

Supplementary Table 5 l Oligonucleotide sequences used for assembly of the multi-strand (msP) control. Note the modules used for the design of msP differ from the set used for 4Pys construction due to the instability of the multi-strand design constructed using the same set of modules. To this end, modules with greater thermodynamic stability were generated for msP construction.

msP_str1	CAGGCCAGGCGCACTTTTCCCGGCCCGGTAGGTTTTCGGGAGGGCGGGCG
msP_str2	CCTACCGGGCCGGGTTTTGTGCCCTGTCCGCCTTTTCGCGTGCGACACGC
msP_str3	GGCGGACAGGGCACTTTTGGTCGCCACGGCAGTTTTGGGGCCTCCTTCGC
msP_str4	CTGCCGTGGCGACCTTTTGTGCGCCTGGCCTGTTTTGGCCGTTGCTCCCC
msP_str5	AACGGCCTTTTCGCCCGCCCTCCCGTTTTGCGTGTCGCACGCGTTTTGCG AAGGAGGCCCCTTTTGGGGAGC

Supplementary Table 6 l Expected fragment lengths resulting from RE digestion of P1's sense (+) and antisense (-) chain. dsDNA controls digestion patterns result in occurrence of additional bands (bold type) that result from digestion of the antisense (-) chain.

P1 digestion	(+) chain	(-) chain
SalI	96, 90, 100	100, 90, **104**
SmaI	116, 108, 62	**66**, 108, **120**
EcoRV	62, 108, 116	**120**, 108, **66**
BamHI	42, 198, 46	46, 198, **50**
EcoRI	132, 126, 28	28, 126, **140**
HindIII	24, 126, 136	136, 126, **32**
BsrGI	6, 72, 208	208, 72, **14**
SpeI	204, 72, 10	10, 72, **212**

Supplementary Table 7 | The order parameter used in FFS simulations for the formation of module *i* in the P0, P1, and P4 4Py designs. The parameter d_i is the minimum distance between any intended base pair in module *i* (as measured from the center of mass of the bases), and x_i is the number of intended base pairs in module *i.*

Q^i	Description
-2	$d_j > 5.1$ nm for all $j \geq i$ & $d_k \leq 5.1$ nm for all $k < i$
-1	0.85 nm $< d_i \leq 5.1$ nm & $d_k \leq 5.1$ nm for all $k < i$
0	$d_i \leq 0.85\ nm$ & $x_i = 0$ & $d_k \leq 5.1$ nm for all $k < i$
1	$x_i \geq 1$ & $d_k \leq 5.1$ nm for all $k < i$
2	$x_i \geq 2$ & $d_k \leq 5.1$ nm for all $k < i$
3	$x_i \geq 6$ & $d_k \leq 5.1$ nm for all $k < i$
4	$x_i \geq 14$ & $d_k \leq 5.1$ nm for all $k < i$

Supplementary Table 8 I Results of FFS for the folding of the P0 pyramid (modules: 1 - 8). The flux was measured for the crossing of $\lambda_{Q^i=-1}^{Q^i=0}$ and probabilities of reaching $\lambda_{Q^i-1}^{Q^i}$ from $\lambda_{Q^i-2}^{Q^i-1}$. Results presented without the standard error of the mean value were obtained from one independent rate calculation.

Module 1

λ	Crossings	Total time (s)	Flux (s^{-1})
λ_{-1}^{0}	17355	0.92e-03	1.88e+07 ± 5.47e+05
λ	**Success**	**Attempts**	**Fractional success**
λ_0^1	9002	828563	0.0106 ± 0.0005
λ_1^2	9010	49439	0.1849 ± 0.0101
λ_2^3	9209	32162	0.2917 ± 0.0119
λ_3^4	5334	5597	0.9538 ± 0.0024
**Total Melting Events:** N/A		k_{14}^{1}/k_{-}^{0}: N/A	

Module 2

λ	Crossings	Total time (s)	Flux (s^{-1})
λ_{-1}^{0}	7286	0.40e-03	1.88e+07 ± 7.49e+05
λ	**Success**	**Attempts**	**Fractional success**
λ_0^1	4001	318777	0.0121 ± 0.0009
λ_1^2	4001	37248	0.0887 ± 0.0134
λ_2^3	4120	30683	0.1098 ± 0.0176
λ_3^4	3192	5759	0.5099 ± 0.0345
**Total Melting Events:** 0		k_{14}^{2}/k_{-}^{1}: N/A	

Module 3

λ	Crossings	Total time (s)	Flux (s^{-1})
λ_{-1}^{0}	7258	0.30e-03	2.45e+07 ± 2.69e+05
λ	**Success**	**Attempts**	**Fractional success**
λ_0^1	4002	347346	0.0110 ± 0.0013
λ_1^2	4002	39262	0.1002 ± 0.0037
λ_2^3	4078	64895	0.0891 ± 0.0351
λ_3^4	3215	4652	0.7094 ± 0.0048
**Total Melting Events:** 251		k_{14}^{3}/k_{-}^{2}: 0.0019	

Module 4

λ	Crossings	Total time (s)	Flux (s^{-1})
λ_{-1}^{0}	7209	0.03e-03	28.7e+07 ± 26.65e+05
λ	**Success**	**Attempts**	**Fractional success**
λ_0^1	4000	230286	0.0176 ± 0.0004
λ_1^2	4008	26447	0.1491 ± 0.0052
λ_2^3	4086	13956	0.3410 ± 0.0756
λ_3^4	2656	2742	0.9669 ± 0.0100
**Total Melting Events:** 27		k_{14}^{4}/k_{-}^{3}: 0.22	

Module 5

λ	Crossings	Total time (s)	Flux (s^{-1})
λ_{-1}^{0}	17242	2.44e-03	0.70e+07 ± 9.98e+05
λ	**Success**	**Attempts**	**Fractional success**
λ_{0}^{1}	9000	861249	0.0110 ± 0.0007
λ_{1}^{2}	9006	78598	0.1102 ± 0.0080
λ_{2}^{3}	9017	498220	0.0194 ± 0.0025
λ_{3}^{4}	5206	685855	0.0092 ± 0.0049
**Total Melting Events:** 230		k_{14}^{5}/k_{-}^{4}: 0.00002	

Module 6

λ	Crossings	Total time (s)	Flux (s^{-1})
λ_{-1}^{0}	12651	0.12e-03	10.40e+07 ± 156.3e+05
λ	**Success**	**Attempts**	**Fractional success**
λ_{0}^{1}	6006	427010	0.0127 ± 0.0024
λ_{1}^{2}	6007	49673	0.1056 ± 0.0431
λ_{2}^{3}	6178	92645	0.0603 ± 0.0333
λ_{3}^{4}	3320	18942	0.1955 ± 0.1053
**Total Melting Events:** 171		k_{14}^{6}/k_{-}^{5}: 0.0005	

Module 7

λ	Crossings	Total time (s)	Flux (s^{-1})
λ_{-1}^{0}	12236	0.09e-03	22.15e+07 ± 929.4e+05
λ	**Success**	**Attempts**	**Fractional success**
λ_{0}^{1}	6002	451631	0.0135 ± 0.003
λ_{1}^{2}	6003	61686	0.0852 ± 0.0282
λ_{2}^{3}	6041	198431	0.0273 ± 0.0143
λ_{3}^{4}	3166	14592	0.2339 ± 0.0374
**Total Melting Events:** 171		k_{14}^{7}/k_{-}^{6}: 0.001	

Module 8

λ	Crossings	Total time (s)	Flux (s^{-1})
λ_{-1}^{0}	12687	0.06e-03	27.38e+07 ± 612.5e+05
λ	**Success**	**Attempts**	**Fractional success**
λ_{0}^{1}	6003	457994	0.0145 ± 0.0018
λ_{1}^{2}	6005	46393	0.1434 ± 0.0190
λ_{2}^{3}	6246	42967	0.1649 ± 0.0263
λ_{3}^{4}	3651	4332	0.8247 ± 0.0516
**Total Melting Events:** 98		k_{14}^{8}/k_{-}^{7}: 0.15	

Supplementary Table 9 | Results of FFS for the folding of the P1 pyramid (modules: 1 - 8). The flux was measured for the crossing of $\lambda_{Q^i=-1}^{Q^i=0}$ and probabilities of reaching $\lambda_{Q^i-1}^{Q^i}$ from $\lambda_{Q^i-2}^{Q^i-1}$.

Module 1

λ	Crossings	Total time (s)	Flux (s^{-1})
λ_{-1}^{0}	20298	1.14e-03	1.78e+07 ± 0.11e+05
λ	**Success**	**Attempts**	**Fractional success**
λ_0^1	10002	758954	0.0133 ± 0.0011
λ_1^2	9166	60279	0.1529 ± 0.0064
λ_2^3	10015	31535	0.3180 ± 0.0136
λ_3^4	4340	4452	0.9748 ± 0.0024
**Total Melting Events:** N/A		k_{14}^1/k_-^0: N/A	

Module 2

λ	Crossings	Total time (s)	Flux (s^{-1})
λ_{-1}^{0}	20299	0.49e-03	4.14e+07 ± 2.23e+05
λ	**Success**	**Attempts**	**Fractional success**
λ_0^1	10001	801699	0.0125 ± 0.0002
λ_1^2	10004	72109	0.1389 ± 0.0044
λ_2^3	10138	56142	0.1806 ± 0.0030
λ_3^4	4222	6721	0.6311 ± 0.0390
**Total Melting Events:** 0		k_{14}^2/k_-^1: N/A	

Module 3

λ	Crossings	Total time (s)	Flux (s^{-1})
λ_{-1}^{0}	20291	0.61e-03	3.30e+07 ± 1.87e+05
λ	**Success**	**Attempts**	**Fractional success**
λ_0^1	10000	773536	0.0129 ± 0.0003
λ_1^2	10007	75121	0.1350 ± 0.0156
λ_2^3	10157	39069	0.2600 ± 0.0017
λ_3^4	4194	5082	0.8253 ± 0.0073
**Total Melting Events:** 60		k_{14}^3/k_-^2: 0.13	

Module 4

λ	Crossings	Total time (s)	Flux (s^{-1})
λ_{-1}^{0}	20352	0.35e-03	5.74e+07 ± 2.99e+05
λ	**Success**	**Attempts**	**Fractional success**
λ_0^1	10003	720073	0.0139 ± 0.0004
λ_1^2	10010	63166	0.1617 ± 0.0228
λ_2^3	10216	35277	0.2898 ± 0.0065
λ_3^4	4128	4324	0.9547 ± 0.0011
**Total Melting Events:** 93		k_{14}^4/k_-^3: 0.14	

Module 5

λ	Crossings	Total time (s)	Flux (s^{-1})
λ_{-1}^{0}	20350	1.02e-03	2.00e+07 ± 7.39e+05
λ	**Success**	**Attempts**	**Fractional success**
λ_0^1	10001	776205	0.0129 ± 0.0004
λ_1^2	10007	75227	0.1343 ± 0.0128
λ_2^3	10181	38454	0.2652 ± 0.0113
λ_3^4	4352	4654	0.9350 ± 0.0028
**Total Melting Events:** 103		k_{14}^5/k_-^4: 0.10	

Module 6

λ	Crossings	Total time (s)	Flux (s^{-1})
λ_{-1}^{0}	20357	0.13e-03	15.6e+07 ± 71.2e+05
λ	**Success**	**Attempts**	**Fractional success**
λ_0^1	10005	588527	0.0170 ± 0.0001
λ_1^2	10004	63481	0.1579 ± 0.0072
λ_2^3	10136	50299	0.2015 ± 0.0022
λ_3^4	4297	6055	0.7108 ± 0.0387
**Total Melting Events:** 23		k_{14}^6/k_-^5: 0.34	

Module 7

λ	Crossings	Total time (s)	Flux (s^{-1})
λ_{-1}^{0}	20236	0.64e-04	31.8e+07 ± 21.4e+05
λ	**Success**	**Attempts**	**Fractional success**
λ_0^1	10001	626969	0.0160 ± 0.0005
λ_1^2	10003	78894	0.1268 ± 0.0010
λ_2^3	10065	57185	0.1784 ± 0.0205
λ_3^4	4169	4554	0.9154 ± 0.0126
**Total Melting Events:** 23		k_{14}^7/k_-^6: 0.29	

Module 8

λ	Crossings	Total time (s)	Flux (s^{-1})
λ_{-1}^{0}	20182	0.67e-04	30.1e+07 ± 56.9e+05
λ	**Success**	**Attempts**	**Fractional success**
λ_0^1	10001	716317	0.0140 ± 0.0002
λ_1^2	10003	85553	0.1177 ± 0.0098
λ_2^3	10029	92218	0.1090 ± 0.0056
λ_3^4	4102	4694	0.8739 ± 0.0020
**Total Melting Events:** 21		k_{14}^8/k_-^7: 0.15	

Supplementary Table 10 I Results of FFS for the folding of the P4 pyramid (module: 1 - 8). The flux was measured for the crossing of $\lambda_{Q^i=-1}^{Q^i=0}$ and probabilities of reaching $\lambda_{Q^i-1}^{Q^i}$ from $\lambda_{Q^i-2}^{Q^i-1}$.

Module 1

λ	Crossings	Total time (s)	Flux (s^{-1})
λ_{-1}^{0}	6359	0.13e-03	4.74e+07 ± 1.63e+05
λ	**Success**	**Attempts**	**Fractional success**
λ_0^1	3000	212475	0.0147 ± 0.0021
λ_1^2	3004	17637	0.1732 ± 0.0158
λ_2^3	3218	8093	0.4041 ± 0.0319
λ_3^4	3464	3530	0.9813 ± 0.0035
**Total Melting Events:** N/A		k_{14}^1/k_{-}^0: N/A	

Module 2

λ	Crossings	Total time (s)	Flux (s^{-1})
λ_{-1}^{0}	6400	0.13e-03	4.99e+07 ± 15.39e+05
λ	**Success**	**Attempts**	**Fractional success**
λ_0^1	3001	271017	0.0111 ± 0.0003
λ_1^2	3005	21709	0.1613 ± 0.0388
λ_2^3	3207	17007	0.1931 ± 0.0214
λ_3^4	3472	3720	0.9335 ± 0.0054
**Total Melting Events:** 0		k_{14}^2/k_{-}^1: N/A	

Module 3

λ	Crossings	Total time (s)	Flux (s^{-1})
λ_{-1}^{0}	6496	0.36e-03	1.81e+07 ± 0.43e+05
λ	**Success**	**Attempts**	**Fractional success**
λ_0^1	3003	208772	0.0145 ± 0.0011
λ_1^2	3002	17673	0.1742 ± 0.0200
λ_2^3	3234	10000	0.3336 ± 0.0383
λ_3^4	3439	3631	0.9476 ± 0.0143
**Total Melting Events:** 4		k_{14}^3/k_{-}^2: 1.32	

Module 4

λ	Crossings	Total time (s)	Flux (s^{-1})
λ_{-1}^{0}	6415	0.41e-03	1.58e+07 ± 2.41e+05
λ	**Success**	**Attempts**	**Fractional success**
λ_0^1	3000	291785	0.0105 ± 0.0010
λ_1^2	3005	23336	0.1292 ± 0.0055
λ_2^3	3096	83958	0.0377 ± 0.0043
λ_3^4	308	142286	0.0021 ± 0.0003
**Total Melting Events:** 21		k_{14}^4/k_{-}^3: 0.97e-4	

Supplementary Note 1

Free-end gluing

Let $w = s_1 s_2 ... s_{2n}$ be a string in which each symbol appears exactly twice. A symbol s appearing twice in w is a dimer composed of two complementary nucleotide modules within a single polynucleotide chain that glue in an antiparallel orientation. We say that a segment s at position i belongs to a free end if at that stage no segment at positions $i, i+1, i+2, ..., 2n$ has been glued yet. Actually, this defines a tail free end. In a similar way one could define the head free end.

Theorem. There exists a sequence of gluings such that at each step at least one segment of a pair being glued belongs to a free end.

Proof. We give a "greedy" gluing algorithm that takes our string w as input and returns the gluing order of pairs with the property that at each gluing, the second occurrence of a segment being glued belongs to a free end. Code in Python:

```
def gluing_order(w):
  first = set()
  last = []
  for s in w:
      if s in first:
          last.append(s)
      else:
          first.add(s)
  return last
```

Note that the algorithm scans the string w from left to right and the same algorithm can be applied also for a scan in the direction from right to the left. Whenever a symbol s appears for the second time, it is appended to the list last. In the end the completed list last determines the gluing order. However, at the stage when the symbol s is being put in the list, none of the positions coming after it have been glued,

and the theorem is proven. Note that we have used only the tail free end in the proof. This means that the result holds even in the case when one of the termini is fixed.

Supplementary Note 2

oxDNA Model

The oxDNA model provides a good representation of DNA features that are relevant for the current study, including duplex melting temperatures, hairpin stability, and the relative flexibility of single strands[2,3]. In order to accurately capture the relative stabilities of modules containing varying GC content we use the sequence-dependent parameterization of oxDNA[3], where the strengths of hydrogen bonding associated with base pairing and the stacking interactions are dependent on the identity of the bases involved.

The oxDNA model does have some simplifications that must be taken into account when comparing results with real DNA. First, the symmetrical helical structure of duplexes in oxDNA means that the model will not accurately capture effects potentially caused by the major and minor grooves on pyramid folding. Second, oxDNA was parameterized to a salt concentration of [Na^+] = 0.5 M, a regime in which the interactions caused by negatively charged nucleotides are strongly screened. Even though the simulations were not run at the same salt concentration as in the experiments, our primary interest is to investigate the efficiency of the folding in the designed folding pathways, which should not be strongly affected by the salt concentration. Lastly, TT stacking in oxDNA is slightly stiffer than in real DNA, which may affect the flexibility of the pyramid at its vertices.

Molecular Dynamics

Kinetic simulations were performed using an Anderson-like thermostat, similar to the one described in appendix A of reference 4. The Newtonian equations of motion for the system are integrated by Verlet integration[5] with a discrete time-step δt, so that the positions, velocities, orientations, and angular

velocities of the nucleotides are recalculated at each time-step. To model Brownian motion, the velocity of each nucleotide is resampled with a probability $p_v = 0.02$ from a Maxwell-Boltzmann distribution at the temperature of the solvent every 103 time steps. The algorithm also resamples angular velocities with a different probability $p_\omega = 0.0067$. On time scales longer than $N_{Newt}\,\delta t / p_v$ the dynamics is diffusive. We choose $\delta t = 1.52 \times 10^{-14} s$ for all dynamics simulations in this study. In oxDNA this time step gives a diffusion constant D_{sim} for a 14-mer duplex that is about 100 times higher than experimental measurements[6] of $D_{sim} = 1.19 \times 10^{-10} m^2 s^{-1}$. This artificial increase in D_{sim} is a common procedure for coarse-grained models where higher diffusion constants can be used to accelerate sampling. The coarse-grained nature of the potential also means the free-energy landscape is, on a microscopic scale, smoother than for real DNA, additionally accelerating dynamic processes[7]. See reference 1 for more details and discussion of D_{sim} in oxDNA. These features make the sampling of complex self-assembly pathways more computationally feasible. Here we are primarily concerned with using simulations to probe the relative rates of similar processes, so the absolute magnitude of the rates is not an issue.

Forward Flux Sampling

We used direct Forward Flux Sampling (FFS) to more efficiently calculate rates between local free-energy minima, as well as sample the transition pathways between minima. Direct FFS is illustrated schematically in Supplementary Fig. 12. Here we summarize the essential points regarding usage of the algorithm. In FFS simulations, we use an order parameter Q^i that measures the progress of the reaction from a macrostate A_{i-1} to a macrostate A_i, where $i = 1...8$ refers to the number of folded modules in that state. The flux Φ_{A_{i-1},A_i} between macrostates A_{i-1} and A_i is taken to be equal to

$$\Phi_{A_{i-1},A_i} = \frac{N_{A_{i-1},A_i}}{\tau f_{A_{i-1}}}, \qquad (1)$$

where N_{A_{i-1},A_i} is the number of times the simulation that reached A_i started in A_{i-1}, τ is the total simulation time, and f_A is the fraction of τ in which the state A_{i-1} was visited more recently than A_k, where $k \neq i-1$.

In the phase space between these states, we draw successive non-intersecting interfaces $\lambda_{Q^i-1}^{Q^i}$ for different values of Q^i. At the start of FFS simulations, an estimate of the flux is measured as the total number of configurations that started in a state described by $Q^i = -2$ that cross λ_{-1}^{0}. The total flux of trajectories from $Q^i = -2$ to the free-energy minimum $Q_{\max}^i$ can be calculated as the flux of trajectories crossing λ_{-1}^{0} times the probability that trajectories reach $Q^i = Q_{\max}^i$ before returning to $Q^i = -2$. The probability can be factorized as

$$P\left(\lambda_{Q_{\max}^i-1}^{Q_{\max}^i} \middle| \lambda_{Q^i=-1}^{Q^i=0}\right) = \prod_{Q^i=1}^{Q_{\max}^i} P\left(\lambda_{Q^i-1}^{Q^i} \middle| \lambda_{Q^i-2}^{Q^i-1}\right). \qquad (2).$$

The first term in the product on the right-hand side of Supplementary Equation 2 is calculated by loading random configurations that have just crossed $\lambda_{Q^i=-1}^{Q^i=0}$, which are used to estimate $P\left(\lambda_{Q^i=0}^{Q^i=1} \middle| \lambda_{Q^i=-1}^{Q^i=0}\right)$. The process is then iterated for successive interfaces, and the flux as well as the trajectories that successfully reach $Q_{\max}^i$ from the distribution of pathways can be sampled.

Mimicking annealing in FFS simulations is not computationally feasible for the current study due to the relatively wide temperature range over which the strand is designed to fold. Thus, all FFS simulations of 4Py designs were carried out at a constant temperature (72°C). In experiments, competing metastable structures are thermodynamically disfavored for P1, where slow annealing encourages the strand to fold according to the designed folding pathway (Fig. 2). Simulations running at a constant temperature may undesirably sample out-of-order folded metastable structures that may be long-lived. To avoid complications caused by metastable structures forming in simulations, we sampled only the folding of modules in the exact same order for a particular design as shown in Fig. 2. Specifically, we prevented all subsequent modules (*i*+1) from forming structure until module *i* formed completely for the first time. This

choice in sampling helped to reduce simulation time, as less statistics needed to be generated in order to sample well the ensemble of folding trajectories of individual modules.

The simulations for the formation of module *i* may also sample melting of other already-formed modules. We keep track of the number of times melting events occur in flux generation simulations, which can be used to estimate melting rates. The estimated melting rates for all studied 4Pys are listed in Supplementary Tables 8-10. Configurations in flux generation simulations which do melt a pre-formed module are taken to have failed and are thus discarded, while configurations used in probability estimation simulations that sample melting of a pre-formed module are taken to have failed while attempting to cross the interface.

We estimated the random error in the FFS simulations in the following way. In Fig. 2 in the main text we reported the mean value for the formation rates of modules from several identical and independent implementations of FFS for the 4Py strands that we considered. The error reported for each 4Py is the standard error of the mean value. In Supplementary Tables 8-10 we report the mean and the standard error of the mean for each individual interface for all 4Py studied. We note that this estimation of the error may be an underestimate of the true variance in the data because we were only able to calculate each rate independently two or three times due to limited computational resources.

Simulations Protocols

In all kinetics simulations we used a simulation box with a volume of $1.33\times10^{-22}m^3$ which corresponds to a concentration of 12.4 μM. All single strand sequences that we studied were simulated at a constant temperature of T = 72°C. To investigate the complete folding of a pyramid, we calculated the formation rates of eight modules forming successively in the designed order for P0, P1, and the first four connection-forming steps in P4. The notation $i=1...8$ refers to the order of intended folding in a particular design, where i = 1 refers to the formation of the first module, and i = 8 refers to the formation of the final module completing a fully formed pyramid.

Order Parameter Used in FFS Simulations

We used two types of order parameters in the simulations, which we combined into one multi-dimensional order parameter. Specifically, a 'distance' order parameter (*d*) measures the minimum distance between hydrogen-bonding sites over correct pairs of bases in the two strands segments in a module. A 'bonds' order parameter (*x*) measures the total number of base pairs in a module. The definition of a bonded base pair in our simulations is two interacting bases with an absolute hydrogen bonding energy smaller than 0.596 kcal mol^{-1}. The value for the selected cut-off corresponds to about 15% of the typical hydrogen-bond energy. Distance criteria are used to define states $Q^i = -2, -1, 0$, and bonding criteria are used to define states $Q^i = 1, 2, 3, 4$ for the formation of the module *i*, respectively. The bond criteria for states $Q^i = 1, 2, 3, 4$ tracks only the number of correctly aligned intra-strand base pairs in module *i,* that is, only those base pairs which are present in the target structure.

In each simulation sampling the formation of module *i*, a configuration was defined to be in a (meta)stable A_{i-1}-state, a structure containing (*i*-1) number of fully formed modules, when all distance parameters d_j for all $j \geq i$ measured greater than 5.1 nm, and all $d_k \leq 5.1$ nm for all $k < i$ (i.e., all pre-formed modules are intact). During the formation of module *i*, a configuration is taken to have reached the A_i-state when the bond parameter x_i measures 14 base pairs for the first time, and all previously formed domains at the start of the rate calculation remained intact throughout the course of the transition. The order parameter details are listed in Supplementary Table 7.

With the order parameter defined, the transition rate for the formation of module *i* in a designed 4Py folding pathway (see Fig. 2), is computed from data obtained from FFS simulations as

$$k_{14}^i = \left(\Phi^i P\left(\lambda_{-1}^0 \middle| \lambda_0^1\right)\right)\left(P\left(\lambda_0^1 \middle| \lambda_1^2\right) P\left(\lambda_1^2 \middle| \lambda_2^3\right) P\left(\lambda_2^3 \middle| \lambda_3^4\right)\right) = k_1^i p_{1\to14}^i, \quad (3)$$

where Φ^i in the first line is the computed flux, λ represents the interfaces for different values of Q^i, k_1^i is the rate of formation of the first correct base pair in module *i*, and $p_{1\to14}^i$ is the probability this base pair leads to the formation of 14 base pairs for the first time, thus completing the formation of module *i*.

Initialization of Single-Strand States for Use in FFS Simulations

FFS requires the initial configurations that are used to start flux generation simulations to be thermodynamically representative of the A_{i-1}-state. However, in simulations sampling the formation of a pyramid, configurations may form 14 bp (our requirement for the complete formation of a module) before the tail has completely threaded. For pyramid systems it was not possible to implement Monte-Carlo simulations, which could be used to calculate relative free energies between states with completely threaded tails and those that are incompletely threaded, because we lack a systematic approach for measuring the linking number of strands at vertices. We chose to run molecular dynamics simulations at 72 °C before the start of a rate calculation for the folding of module *i* in an attempt to relax trial configurations. The simulations sample, in addition to any pre-formed pyramid structure, only the diffusion of unbound single stranded regions, which are prevented from forming any unintended base pairs that could lead to competing metastable structures. Since the tails only need to sample their local environments, which we naively expect they will be able to do with relative ease, the barrier between threaded and incompletely threaded tails should not necessarily be exceedingly large for the P1 system, where the longer tails have to thread when less pyramid structure is in place. Simulations that together accumulate a long amount of simulation time should be sampling at least some representative states. The relaxation simulations may also sample the melting of a previously formed module. Configurations that do lose modules were collected but were not used in any future simulations. To obtain a set of relaxed configurations, 500 starting configurations were randomly selected from the final state of the previous rate calculation for the formation of the module (*i*-1), where each configuration was simulated for 0.152 μs before being saved. A configuration from this relaxed set is selected at random and set to be the starting configuration in a flux generation simulation.

Measurement of the Height of P1

As reported in the main text, we measured the average height $\langle h \rangle$ of P1 in simulations after cooling completely folded configurations down to 25°C. The average height was predicted to be 4 ± 0.3 nm. In Supplementary Fig. 13 the geometry of a pyramid, and relevant points and vectors that were used in calculating the height, are illustrated. Since the pyramid is rather flexible, we constructed vectors normal to the base of a pyramid at a vertex *i* as $n_i = v_{i,2} \times v_{i,1}$ where $v_{i,1}$ and $v_{i,2}$ are vectors representing the displacement of the two modules meeting at the vertex *i*. We measure the height from the point-of-view at vertex *i* to be the distance between the centre of mass of the linker bases at the top of the pyramid, represented by the point T, to a projected point PT_i on the plane defined by n_i at the point X_i, which we take to be the centre of mass of the base pair in the middle of one of the modules adjacent to the vertex *i*. That is, $h_i = |PTT_i|$ which is measured four times per configuration. The average height reported in the main text was calculated by running 5 independent molecular dynamics simulations, which each ran for 7.6 μs and saved configurations every 1.52 ns. The height measurements for each configuration in a trajectory were computed and averaged together to obtain an average value for the trajectory. The reported average $\langle h \rangle$ and the error were calculated as the mean and the standard error of the mean of the independent simulations.

Kinetics Results

The cumulative statistics of the FFS simulations for the complete formation of P0 are listed in Supplementary Table 8**,** for the P1 system in Supplementary Table 9, and the formation of the first four connection-forming steps in the P4 system in Supplementary Table 10.

Supplementary Tables 8-10 also list the estimated melting rates of a module (k_-^{i-1}) which were sampled during the simulations for the formation of module *i*. The melting rate of a module is estimated by dividing the total number of sampled melting events, which occurred during flux generation simulations, divided by the total simulated flux time. For the module formation events that were sampled in P1, all estimated

melting rates are larger than the corresponding calculated forward rate by at most a factor of ten. P4 is less affected by melting until the first kissing hairpin forms, at which point module formation becomes extremely slow. P0 is also affected by melting of pre-formed modules, and is most prominent during the sampling of module formation when long tails must thread loops for the module to be completely formed.

There are two main reasons for the high rate of melting of pre-formed modules. First, pyramids containing improperly threaded modules are much more likely to melt because the pyramid is rather flexible at its vertices due to the linkers, which can help relieve the stress caused by a topological defect by allowing the affected module to lose some base pairs and partially unwind. Secondly, the simulation temperature is in the vicinity of the melting temperature of the modules, so properly formed modules may melt spontaneously. Most melting events in P0 and P1 are due to modules 2 and 3 unfolding, which are also the modules that we observe most often containing an improperly threaded tail. However, not all long tails lead to a higher incidence of defective modules in completed pyramids. For example, in the P0 system we see module 5, another double helix that forms as a result of a tail containing three modules threading a loop, forming correctly in the majority of sampled pyramids. The higher probability of forming defect-free modules is caused by a comparably smaller loop that the long tail must thread in order to form all base pairs when compared to the formation of module 3 in P1, because more pyramid structure is in place.

As discussed in section “Order Parameter Used in FFS Simulations”, the implemented order parameter could not tell us whether a tail had completely threaded or not. Consequentially, not all of the initial simulations run prior to FFS simulations of the formation of module *i* may have sampled thermodynamically representative configurations of the state A_{i-1}. However, since we were able to sample pyramids with and without defects in several folding pathway designs, incomplete threading of modules may not be strongly favored over completely threaded tails in the final simulated structure. Thus, the rates of formation of modules may only be marginally affected by the formation of topological defects. However melting rates are likely to be over-estimated. Simulations at lower temperatures would suppress the high rate of melting but may not help much in reducing the number of topologically defected modules, which are likely to still be present regardless of the simulation temperature. While there is uncertainty in

the calculated relative transition rates that we report due to these factors, the general features of the sampled transition pathways discussed in the main text are preserved.

Supplementary References

1 Ouldridge, T. E., Sulc, P., Romano, F., Doye, J. P. & Louis, A. A. DNA hybridization kinetics: zippering, internal displacement and sequence dependence. *Nucleic Acids Res.* **41,** 8886-8895 (2013).

2 Doye, J. P. K. *et al*. Coarse-graining DNA for simulations of DNA nanotechnology. *Phys. Chem. Chem. Phys.* **15,** 20395-20414 (2013).

3 Sulc, P. *et al*. Sequence-dependent thermodynamics of a coarse-grained DNA model. *J. Chem. Phys.* **137,** 135101 (2012).

4 Russo, J., Tartaglia, P. & Sciortino, F. Reversible gels of patchy particles: role of the valence. *J. Chem. Phys.* **131,** 014504 (2009).

5 Verlet, L. Computer "Experiments" on Classical Fluids. I. Thermodynamical Properties of Lennard-Jones Molecules. *Phys. Rev.* **159,** 98-103 (1967).

6 Lapham, J., Rife, J. P., Moore, P. B. & Crothers, D. M. Measurement of diffusion constants for nucleic acids by NMR. *J. Biomol. NMR* **10,** 255-262 (1997).

7 Murtola, T., Bunker, A., Vattulainen, I., Deserno, M. & Karttunen, M. Multiscale modeling of emergent materials: biological and soft matter. *Phys. Chem. Chem. Phys.* **11,** 1869-1892 (2009).
